# Thermosensory Spiking Activity of Proteinoid Microspheres Cross-Linked by Actin Filaments

**DOI:** 10.1021/acs.langmuir.4c01107

**Published:** 2024-06-05

**Authors:** Panagiotis Mougkogiannis, Andrew Adamatzky

**Affiliations:** Unconventional Computing Laboratory, UWE Bristol, Bristol BS16 1QY, U.K.

## Abstract

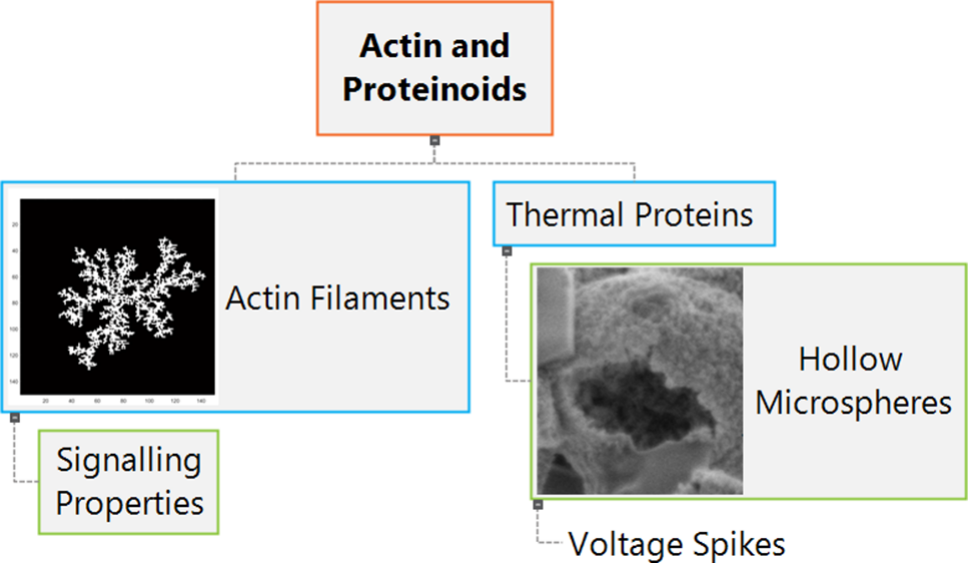

Actin, found in all eukaryotic cells as globular (G) or filamentous (F) actin, undergoes polymerization, with G-actin units changing shape to become F-actin. Thermal proteins, or proteinoids, are created by heating amino acids (160–200 °C), forming polymeric chains. These proteinoids can swell in an aqueous solution at around 50 °C, producing hollow microspheres filled with a solution, exhibiting voltage spikes. Our research explores the signaling properties of proteinoids, actin filaments, and hybrid networks combining actin and proteinoids. Proteinoids replicate brain excitation dynamics despite lacking specific membranes or ion channels. We investigate enhancing conductivity and spiking by using pure actin, yielding improved coordination in networks compared with individual filaments or proteinoids. Temperature changes (20 short-peptide supramolecular C to 80 °C) regulate conduction states, demonstrating external control over emergent excitability in protobrain systems. Adding actin to proteinoids reduces spike timing variability, providing a more uniform feature distribution. These findings support theoretical models proposing cytoskeletal matrices for functional specification in synthetic protocell brains, promoting stable interaction complexity. The study concludes that life-like signal encoding can emerge spontaneously within biological polymer scaffolds, incorporating abiotic chemistry.

## Introduction

Actin is a protein found in all eukaryotic cells, existing in the forms of globular actin (G-actin) and filamentous actin (F-actin).^1−3^ G-actin polymerizes into a double helix of filamentous actin, with G-actin units undergoing slight shape changes during polymerization to become F-actin units.^4^ Actin networks play a crucial role in information processing within living cells.^5−8^ Actin networks exhibit electrical signaling and conduction due to interactions between charged biopolymers and mobile ions.^9−16^ Atomistic simulations show that actin filaments display soliton propagation and nonlinear capacitive properties influenced by voltage inputs and molecular conformations.^17^ These dynamics impact signal transmission through cytoskeletal structures. Studies with 3D actin architectures reveal their ability to conduct electrical currents and be metalized for electronic connections.^18^ Bottom-up self-organization and top-down micropatterning enable precise construction of conductive channels. Cortical actin layers in cells regulate electroporation kinetics, with disruption under high voltage settings. Biomimetic experiments shed light on the cytoskeleton’s role in cellular electropermeabilization.^19^ Computational investigations indicate that abstracted actin bundle networks can execute logic operations, storing states, and responding to stimuli.^20^ These diverse capabilities, from biophysical signaling to artificial computation, result from cooperative interactions exploiting actin networks’ natural excitability.^21^ Actin filaments play a critical role as structural scaffolds in neuronal excitability, actively contributing to various functions, including providing structural support and modulating synaptic activity.^6,22^

Table 1 details numerous biological polymers that share similarities with actin in terms of functions, including membrane shape, cross-linking, and supporting ion channel coupling crucial for coordinating electrical signaling processes. Microtubules aid in long-distance axonal transport,^34^ while spectrin regulates the movement of transmembrane proteins at specific locations.^28,35^ Intermediate filaments provide mechanical stabilization for force transmission, whereas cell adhesion molecules play a crucial role in promoting the formation of synaptogenic connections.^36,37^ The complex interaction between cytoskeletal proteins, adapter molecules, and adhesion proteins demonstrates the wide range of tools cells use to control excitability.^38^ Bioinspired materials aim to mimic the complex biophysics of neurons by replicating conductive scaffolds and creating integrated systems that facilitate hierarchical assembly. This study investigates the use of proteinoid microspheres in actin filaments to create electroactive networks, aiming to mimic the complexities of neuronal activity through intentional design interventions. Previously, we demonstrated the implementation of Boolean, multivalued, and quantum logical gates on coarse-grained models of actin filaments using cellular automata, quantum automata, and a lattice with Morse potential approaches.^39−43^ Theoretical designs of actin-based logical circuits achieve logical gates through collisions between traveling localizations, assuming precise control over nearly every atom in the actin molecule^44^ or the exact timing of collisions between traveling localizations.^43^ In our theoretical models,^45,46^ we considered actin as a substrate in which signals (traveling localizations) interact and perform Boolean gates by the interaction. However, there was no indication of how the signals were generated. This is how proteinoids came to light.

**Table 1:** Actin Filament Analogues and Neuronal Signaling Functions

scaffold	related cytoskeletal features	neuronal signaling role
microtubules^23,24^	structural biopolymer	axonal transport, growth cone migration
intermediate filaments^25,26^	cytoskeletal protein	mechanical stabilizer, kinase scaffolding
spectrin^27,28^	cross-linking protein^29,30^	ion channel tethering, synapse scaffolding
CAMs^31,32^	cell adhesion molecule	synaptogenesis, neurite outgrowth
Tcams	cell adhesion transmembrane protein^33^	synapse assembly, receptor clustering

Proteinoids, or thermal proteins, are formed by heating amino acids to their melting point, initiating polymerization to form polymeric chains. This process occurs at 160–200 °C without any solvent, initiator, or catalyst, within a neutral environment. Certain amino acids, such as glutamic acid, aspartic acid, or lysine, serve as solvents and initiators for the polymerization of other amino acids through cyclization at high temperatures.^47,48^ The simple thermal condensation reaction allows the production of either acidic or basic proteinoids. These proteinoids can form microspheres when swollen in an aqueous solution at moderate temperatures (approximately 50 °C), creating structures known as microspheres^48^ (Figure 2D), which are typically hollow (Figure 3) and filled with an aqueous solution. Microsphere growth is programmable, with sizes ranging from 20 to 200 μm, controlled by the selection of amino acid subsets and thermal regimes.^48^ Proteinoid microspheres maintain a steady-state membrane potential of 20–70 mV without external stimulation. Some microspheres exhibit a steady opposite polarization.^49^ Electrical membrane potentials, oscillations, and action potentials are observed in microspheres impaled with microelectrodes, displaying action-potential-like spikes. This electrical activity includes spontaneous bursts of electrical potential (flip-flops) and miniature potential activities during flopped phases.^50^ In 20 μm microspheres, the amplitude is 20 mV, while in 200 μm microspheres with lecithin, it reaches 70 mV. The amplitude of spiking is regular in phospholipid-free microspheres.^51^ Membrane, action, and oscillatory potentials recorded from microspheres composed of thermal protein, glycerol, and lecithin^50,51^ are observed for several days.^52^ The microspheres remain stable^53^ in water at pH levels above 7.0 and continue oscillating for weeks.^49^

In short, we aimed to design and implement protobrain ensembles of proteinoid microspheres (proto-neurons) connected with each other with actin filaments (protoneural terminals).

Dynamic electrical activity and conduction are not limited to complicated biological systems; they can occur spontaneously in simpler abiogenic polymers, such as proteinoid microspheres. Cell-like units are produced by assembling heated amino acid blends, which display action potential spikes, membrane oscillations, and environment-dependent behavior.^50,54^ These life-like occurrences emerge naturally, without the requirement for specific proteins or evolutionary adaption. Furthermore, interconnected proteinoids can create networks with interactive behavior and coordinated reactions,^54^ indicating a type of primordial biotic communication. Furthermore, similar excitability has been discovered in entirely synthetic systems,^55^ indicating that the capacity for electrical activity is not limited to certain biological components. Even hypothesized protocells from the early phases of life could have used light-sensitive electrochemical gradients to drive critical processes.^56−59^

**Figure 1:**
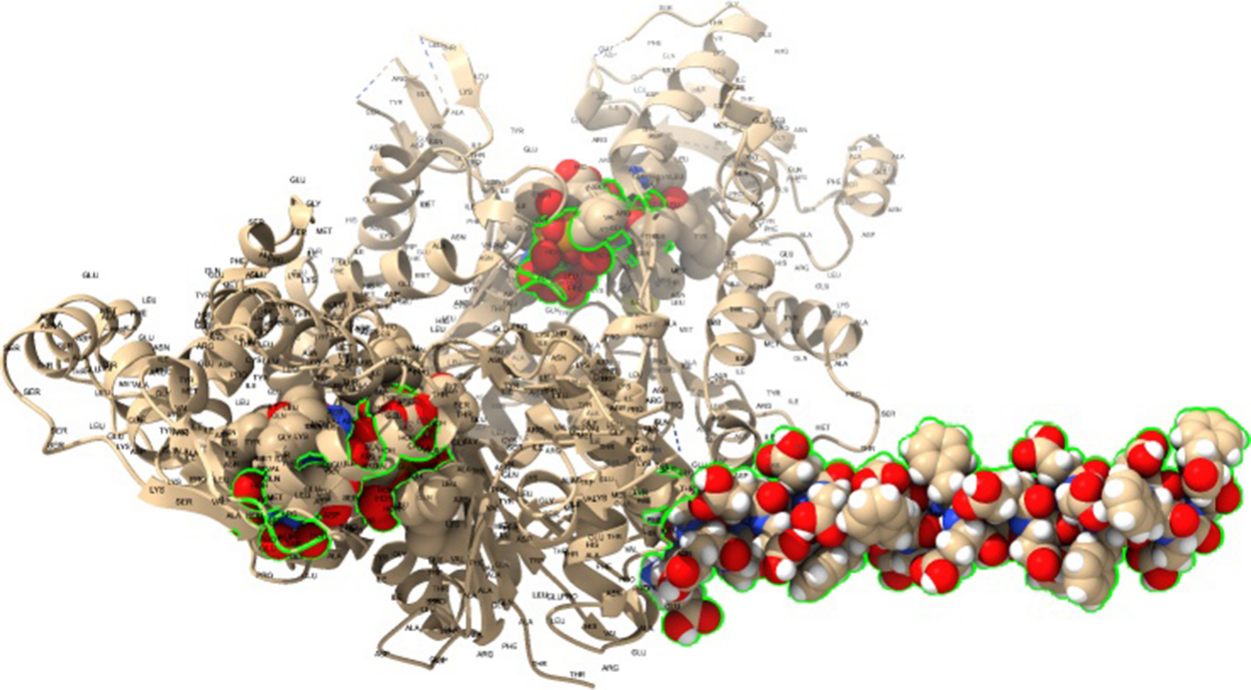
Model demonstrates the interaction between a rabbit muscle actin filament and a heated l-Glu:l-Phe:l-Asp proteinoid. The actin filament has a helical structure made up of globular actin subunits. One end of the filament is connected to the proteinoid membrane interface, indicating possible attachment points for bioconjugation, including surface carboxyl groups. The structures consist of backbone traces and atoms color-coded for both components. Studying the structural features at the proteinoid–actin interface may aid in understanding the relationship between conformational alignment and the activation of conduction channels by the composite. The actin filament’s structure was initially identified and further refined using Protein Data Bank (PDB) data from Xue et al.^60^

Conducive polymer systems allow for biocompatible transmission, detection, and control of cellular networks by connecting abiotic films to endogenous ion fluxes and voltage gradients.^61,62^ This fusion of living organisms and artificial components synergistically combines the various emergent behaviors of living matter with the manipulability and adaptability of synthetic materials. Ongoing research aims to combine life-like proteinoids into modular polymer matrices, bringing us closer to realizing true biomimetic computing systems. These studies aim to combine the numerous functions of proteinoids with the processability and tunability of synthetic materials to establish advanced interfaces between biological and artificial organisms. Figure 1 illustrates the attachment of the proteinoid to the actin filament.

This study investigates the conductive and excitable capacities resulting from the combination of biological cytoskeletal components and synthetic protocell mimetics. A key focus is on quantitatively investigating the bioelectrical behaviors evoked in composite architectures made up of pure actin filament scaffolds and proteinoids (TP) under various production and measurement settings. Systematic multielectrode recordings of electrical activity aim to map the complex dynamical landscape across time scales, capturing both rapid spike occurrences and longer-term oscillatory epochs.

## Result and Discussion

### Morphological Characteristics

**Figure 2:**
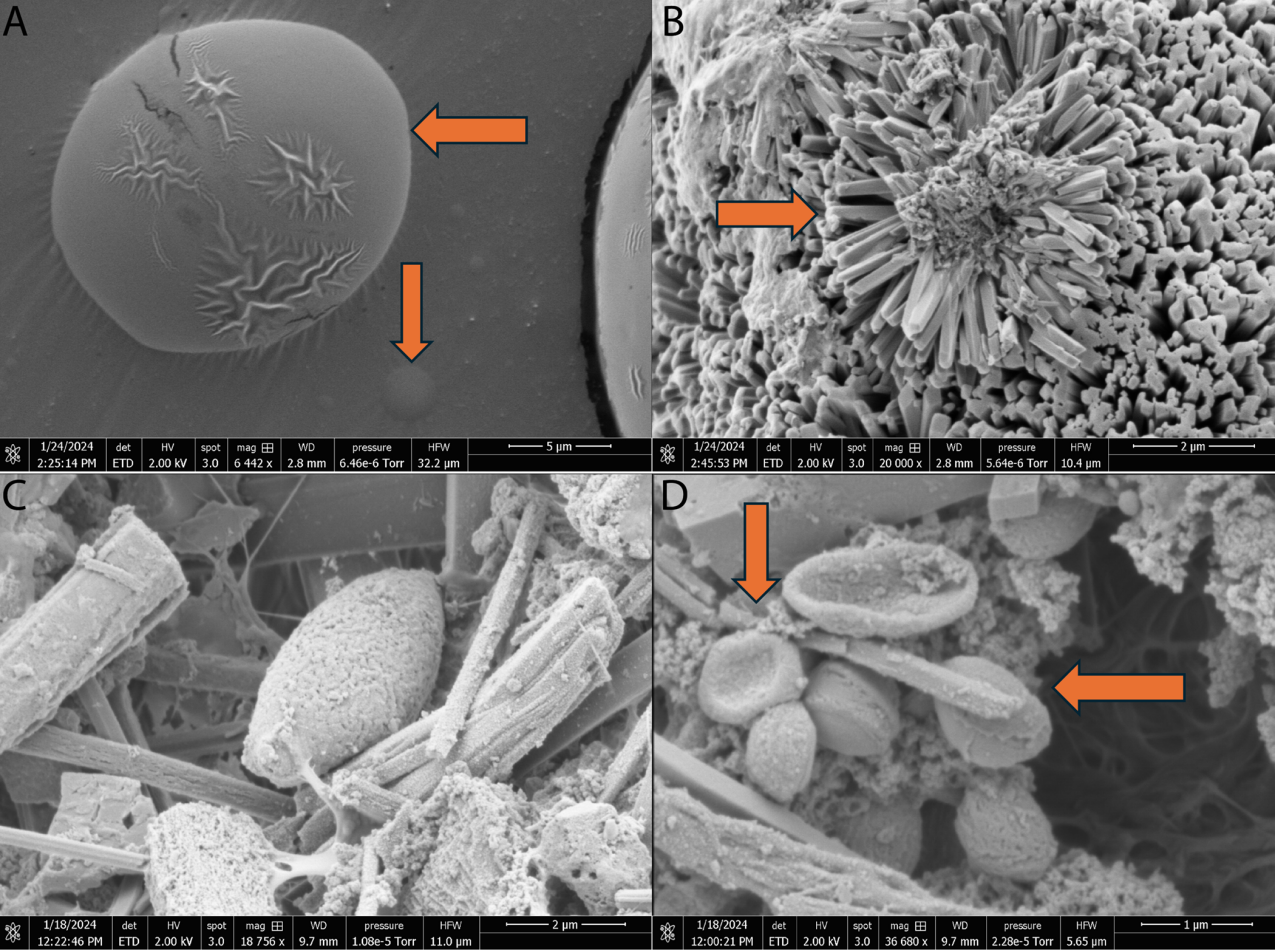
Scanning electron micrographs at various magnifications show the morphology of actin networks and proteinoid microspheres. (A) Actin gel penetration at the membrane interface restricts polymerization in a spherical actin geometry. Arrowheads indicate the presence of actin microspheres, which transform actin into rigid rods and cause actin filaments to form strong bundles. (B) Cross-linked cytoskeletal matrix with actin filaments (arrows) joined by biomolecules (scale bar: 2 μm, 20,000×). (C) Small neural-like ganglion structure formed from proteinoids (scale bar: 2 μm, magnification 18,756). (D) Higher-resolution imaging reveals intricate proteinoid spheres’ aggregated interconnectedness (scale bar: 1 μm, 36,680×). Multiscale visualization reveals a complex morphology, allowing for dynamic emergent functionality ranging from nanoscale structure to mesoscale assembly. The presence of elongated aggregates of actin filaments (arrowheads) with spherical microspheres indicates the existence of interactions between these two components. Using multiscale visualization techniques, intricate morphology is unveiled, enabling the observation of dynamic emergent functionality across a wide range of scales, from the nanoscale structure to the mesoscale assembly. The conversion of tubulin into inflexible rods and the formation of robust actin filament bundles in the presence of actin microspheres suggest an advantageous interaction between the two elements. This interaction potentially enhances the structural stability and functional aspects of the actin–proteinoid composite. Similarly, the elongated appearance of the actin filament clusters implies a strong interconnection and potential cross-linking between them. This aggregation and interconnectedness may play a role in the emergent properties and behavior of the composite system.

Multiscale scanning electron microscopy reveals morphological differences between pure actin scaffolds and the alterations caused by proteinoid (l-Glu:l-Phe:l-Asp) integration (Figure 2A). Cross-linked actin filament matrices (Figure 2B) enable 3D connectivity, allowing signaling to extend beyond individual components. Incorporating proteinoids limits polymerization at phase borders (Figure 2C), thereby defining compartments. Within these boundaries, self–organized neural–like clusters arise (Figure 2C), which then resolve into densely interconnected spheres at greater magnifications (Figure 2D). The observed topological reconfiguration supports significant transitions from homogeneous cytoskeletal networks to the formation of functional biomechanical components via hierarchical reorganization.

The biocomposite matrix is composed of a complex network of actin filaments linked with proteinoid microspheres formed through heat. The main function of the actin component in this composite matrix is to create a structural framework similar to a cytoskeleton. The elongated helical polymer chains in the enlarged 3D meshwork facilitate signal transmission over micrometer-scale distances. These signals can travel either along individual filaments or horizontally through connections between neighboring filaments. Integrating proteinoids into the biocomposite structure generates a transformative shift in the matrix topology, consequently affecting a hierarchical restructuring process. During heat polycondensation, amino acid precursors self–assemble spontaneously to form spherical peptide structures. The organization process is determined by the encoded structure in the matrix. Proteinoids interacting with actin polymers inhibit filament development at the junction, promoting a compartmentalized structure (Figure 2C,D). This results in clearly defined areas where sensor-conductor components are densely concentrated, with their organization controlled by the bottom-up structure of both biological and synthetic interactive building blocks. Complex synaptic-like clusters form in these phase-separated regions due to the affinity connections between actin and proteinoids. Further incorporating proteinoids increases the complexity of these compartments, reinforcing connection through similar fractal–like assembly. The end result of these intricate assembly steps involves arranging nanospheres into cores with concentric layers, including satellite spheres seated tightly within common bioorganic solvent shells.

**Figure 3:**
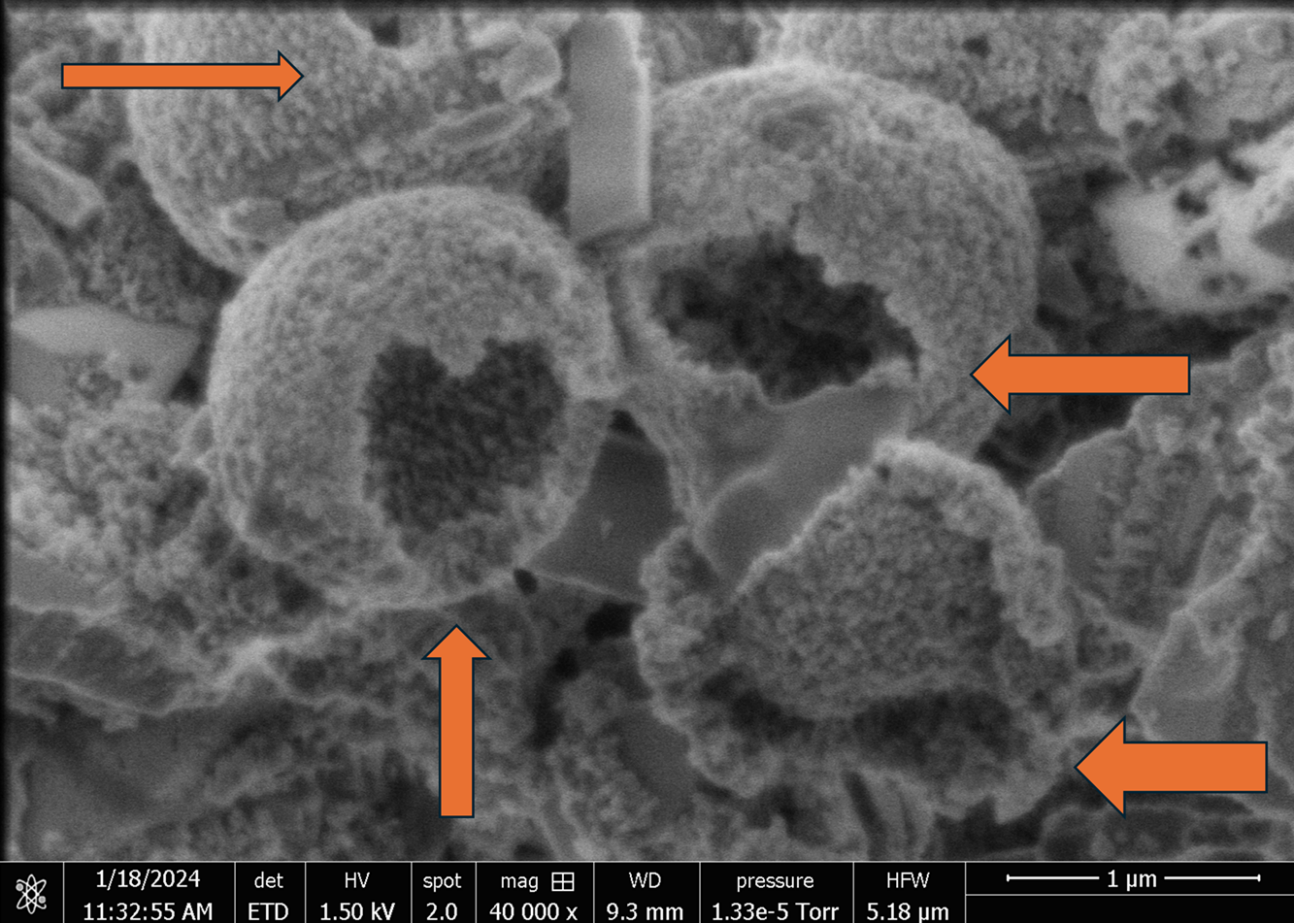
Scanning electron microscopic image showing the structure of hollow proteinoid microspheres that formed when an inert electrolyte (KNO_3_) was present at an ionic strength of 0.065 mol/L. The microspheres display a noticeable hollow configuration (arrowheads), as indicated by the evident contrast between the external shell and the internal cavity. The existence of a hollow interior implies that these structures could be more precisely characterized as proteinoid capsules or vesicles, rather than solid microspheres. The hollow microspheres are formed through the self-assembly of proteinoid molecules under specific ionic conditions. This process results in the formation of a semipermeable membrane that surrounds an aqueous core. This unique architecture may play a crucial role in the electrical properties and potential applications of proteinoid microspheres. Hollow microspheres possess a higher surface area-to-volume ratio in comparison to solid microspheres. This characteristic has the potential to improve their capacity to interact with the surrounding environment and facilitate the transfer of ions and molecules over the membrane. Additionally, the hollow structure may provide a confined space for chemical reactions or the encapsulation and release of active compounds, making them attractive candidates for drug delivery and biosensing applications. Scale bar: 1 μm.

Scanning electron microscopy (Figure 3) verified the formation of hollow proteinoid microspheres when an inert electrolyte (KNO_3_) was present. The micrograph clearly shows a noticeable difference in shape between the outer shell and the inside cavity of the microspheres, which serves as evidence of their hollow structure. The formation of the microspheres occurred when the ionic strength of the proteinoid solution was 0.065 mol/L, indicating that the self-assembly of proteinoid molecules into hollow structures relies on specific ionic conditions. Given this observation, it may be more appropriate to refer to these structures as “proteinoid capsules” or “proteinoid vesicles” rather than “proteinoid microspheres.” These “proteinoid capsules” could have significant implications for their electrical characteristics and potential applications. Hollow microspheres have a greater surface area-to-volume ratio than solid microspheres, which allows them to better interact with the surrounding environment and improve the exchange of ions and molecules across the membrane. Moreover, the empty interior can serve as a restricted area for chemical reactions or the encapsulation and release of active compounds, making them desirable options for drug delivery and biosensing applications. However, further research is necessary to fully comprehend the specific mechanisms that lead to the formation and operation of these hollow proteinoid microspheres and to investigate their superiority over solid microspheres in various scenarios.

### Electrical Activity in Pure Actin Networks

**Figure 4:**
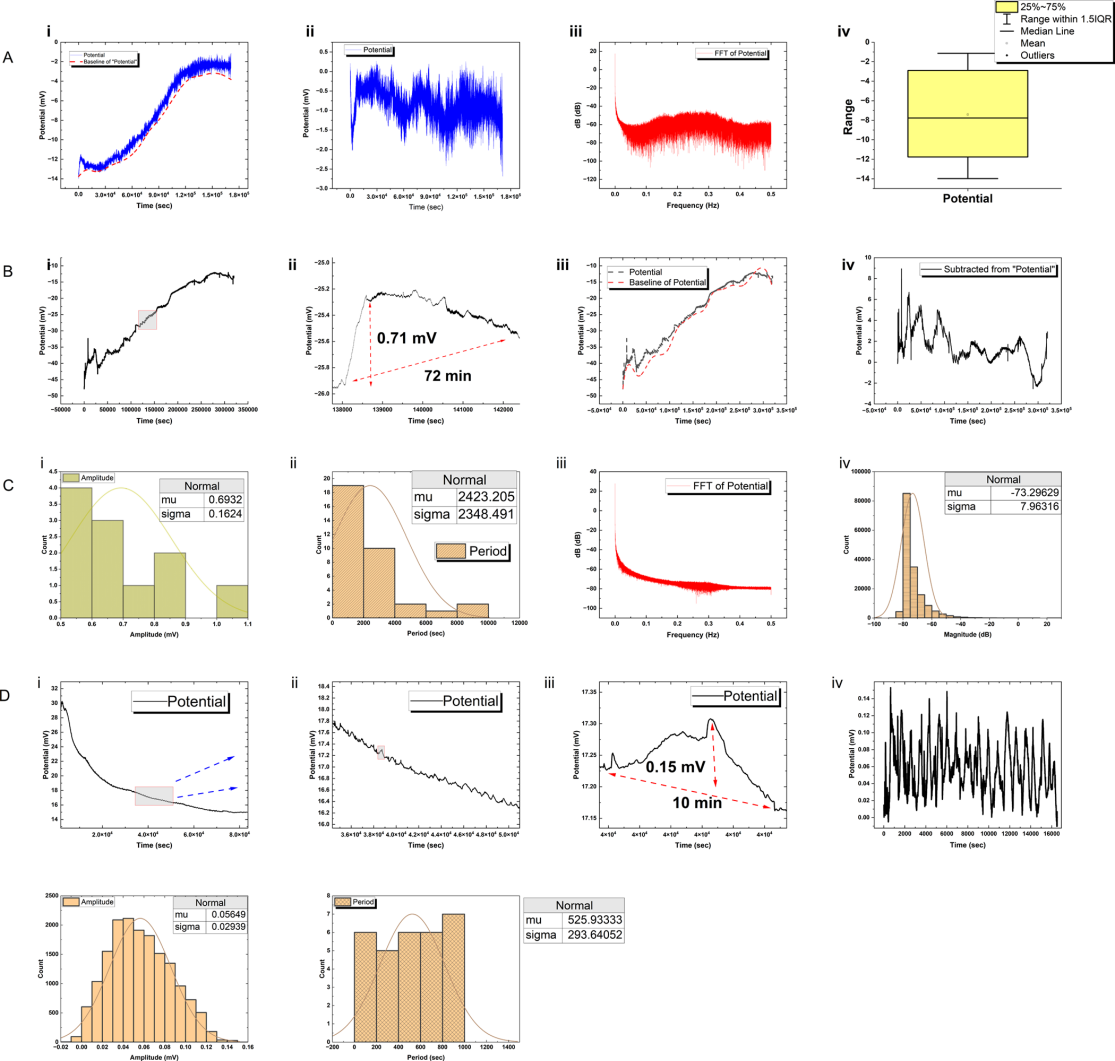
Detailed electrical activity analysis of biomaterial systems. (A) Actin–proteinoid composite networks exhibit episodic bursting events with spike amplitudes up to 0.15 mV and a periodicity of 10 min. (B) Electrical excitation in proteinoid networks shows intermittent spike bursts lasting 11–87 min, with a median duration of 49 min. (C) Signaling patterns of l-Glu:l-Phe:l-Asp proteinoids display normal distribution of spike amplitudes (mean 0.69 mV) and spike period (mean 2423.2 s) with low-frequency outburst signatures. (D) Repeat analysis of actin–proteinoid composite networks confirms structured signaling emergence and coordinated firing events.

A preliminary look at the recorded electrical activity shows that the signals are complex, with bursts and isolated excitation peaks riding on fluctuating baseline potentials. (Figure 4A). Example voltage waveforms over time (Figure 4Aii) showcase transient spikes exceeding underlying offset levels. To emphasize these episodic events, baseline subtraction (Figure 4Aii) highlights the spike morphology and amplitude distributions. Quantitative characterization indicates spikes spanning heights up to 14 mV, with a mean peak excitation of 7.4 ± 4.1 mV emerging from a median background potential of −7.8 mV between events.

An examination of recorded voltage waveforms using frequency analysis offers valuable insights into the primary patterns of bioelectrical activity within the interconnected networks of actin filaments. Figure 4Aiii demonstrates that the power spectral density analysis exhibits a distinct peak at a frequency of 5 × 10^–6^ Hz. This illustrates that global signaling behaviors are driven by bursting patterns and episodic oscillations, rather than solitary events, with a prevalence of low-frequency content. Measuring the distribution of magnitude for specific frequencies (Figure 4Aiv) allows for distinguishing between signal and noise, thereby identifying genuine coordinated occurrences as opposed to random variations. The recorded spectra display an average power of −62.11 dB throughout the recorded duration, with the highest and lowest bandlimited power being +17.41 and −110.29 dB, respectively, at different frequencies. Nevertheless, the maximum strength extends to about 130 dB, indicating that organized activities greatly surpass the background noise, despite variations in frequencies. The spectral magnitude has a standard deviation of 7.54 dB, which indicates that the dynamic range fluctuates significantly across different frequency bands, even when far away from the prominent 5.85 × 10^–6^ Hz activity. This phenomenon refers to the intricate redistribution of spectral intensity throughout periods of heightened and reduced activity when various signaling patterns emerge and fade away through interactions within networks ranging from microscopic to mesoscale levels.

Electrical recordings can be analyzed using spectra analysis to get insights into the distribution of signaling behaviors and noise profiles. As indicated in Table 2, the prevailing power at 5.85 × 10^–6^ Hz provides confirmation that the activity is driven by bursting oscillations rather than discrete spikes. The amplitude of the frequency-band-specific dynamic range, which exceeds 110 dB, suggests that organized events are very distinguishable from the baseline noise. A standard deviation of 7.54 dB indicates that the variability in signal intensity is influenced by the specific spectrotemporal block. Furthermore, examination of the peak electrical potential ranges (Table 3) demonstrates a predominant concentration in the range of −7 to −8 mV, with a variation extending up to 14 mV. Additionally, the presence of the lowest 25% quantile below −11 mV indicates the existence of asymmetric excitation peaks that are distinguished by sudden spike depolarizations followed by gradual recovery. Furthermore, the distribution exhibits intricacy that goes beyond simple binary “on–off” signaling, displaying a wide range of intermittent activations with varying degrees of intensity. In conclusion, the measured spectral and electrical properties offer a detailed analysis necessary for understanding the functional signaling capacities of the dynamic actin filament networks. The experiments provide precise validation of statistically significant synchronized excitability that supports the propagation of self-organized bioelectrical events.

**Table 2:** Power Spectral Density Quantitative Analysis of Baseline Electrical Fluctuations in Actin Filament Matrices and Proteinoid Microspheres^a^

metric	actin	proteinoids
dominant Frequency (Hz)	5.85 × 10^–6^	1.56 × 10^–6^
mean magnitude (dB)	–62.11	–73.30
maximum magnitude (dB)	17.41	27.78
minimum magnitude (dB)	–110.29	–95.20
standard deviation of magnitude (dB)	7.54	7.96

a The summary metrics consist of the dispersion of spectral intensities (measured as standard deviation), the dominant frequency, and the mean and extreme values of magnitude spanning the entire frequency spectrum. Under controlled conditions, the negligible dominant frequency indicates predominantly stochastic dynamics as opposed to periodic patterns. On the contrary, detectable signals exceed the background by approximately −110 dB and are highly concentrated in the sub-1 Hz frequency range. Proteinoids exhibit decreased average signal intensities with increased variability, covering a wider range of activity up to 27 dB signal above the background. This suggests a wider range of behaviors from various structures and adjustable active sites. Although the average and lowest magnitudes are found in extremely low-frequency noise levels below 1 Hz, enhanced peak reactions compared with actin indicate a higher sensitivity to outside interference.

**Table 3:** Summary Statistics of Electrical Potential in Actin Networks, Proteinoids Formed from l-Glu, l-Phe, and l-Asp, and Actin-Cross-Linked Proteinoid Networks under Controlled Conditions (20 °C)^a^

metric	actin	l-Glu:l-Phe:l-Asp proteinoids	actin–proteinoid networks
Potential (mV)			
mean	–7.42	–24.50	18.17
standard deviation	4.15	9.909	3.73
minimum	–13.97	–47.84	–20.57
maximum	–1.14	–12.06	30.27
25% quantile	–11.77	–34.47	15.55
50% quantile (median)	–7.77	–22.72	16.90
75% quantile	–2.90	–14.89	19.30
Spike Amplitude (mV)			
mean	0.07835	0.6317	0.06
standard deviation	0.082	0.2055	0.0294
minimum	–0.1848	0.288	–0.01
maximum	0.2665	1.03	0.15
25% quantile	–0.1447	0.5530	0.03
50% quantile (median)	–0.0960	0.6400	0.05
75% quantile	–0.0296	0.7330	0.08
Spike Period (s)			
mean	36.70	3592.20	520.67
standard deviation	450.23	1030.87	301.77
minimum	3.00	2488.00	15.00
maximum	4952.00	5185.00	941.00
25% quantile	3.00	2706.00	288.00
50% quantile (median)	4.00	3166.50	498.00
75% quantile	7.00	4548.00	764.00
Spectral Power (dB)			
dominant frequency (Hz)	5.85 × 10^–6^	1.56 × 10^–6^	6.05 × 10^–6^
mean magnitude	–62.11	–73.30	–74.99
maximum magnitude	17.41	27.78	24.57
minimum magnitude	–110.29	–95.20	–87.00
standard deviation magnitude	7.54	7.96	7.93
25% quantile magnitude	–66.76	–78.71	–80.29
50% quantile (median) magnitude	–62.14	–75.95	–77.95
75% quantile magnitude	–56.97	–70.93	–72.57

a Key distributional characteristics, such as the mean, variation, and quantiles of the recorded potential difference values, are summarized. Actin and proteinoids exhibit hyperpolarized signals relative to the electrode and overall chaotic fluctuations, with proteinoids showing more variable fluctuations and a broader range of activity. Actin-cross-linked proteinoids networks display transient depolarization events (spikes) with amplitudes fluctuating between 16 and 17 mV above the baseline, and periods ranging from 15 to 941 s. Spectral power distribution reveals bursting oscillations propagating due to a dominant 6.05 × 10^–6^ HZ frequency component, with significant emergent structure and concentrated dynamics around the median −77.95 dB power. Collectively, these metrics provide comprehensive numerical characterization of the electrical signatures of endogenous actin, proteinoids, and their composite networks.

### Electrical Signaling in Proteinoids Systems

Quantitative analysis of recorded waveforms reveals rich dynamical behaviors exhibited by the proteinoids networks. As shown in Table 3, electrical potentials demonstrate a mean spike height of 24.5 mV spanning over 30 mV, indicative of significant coordinated depolarizations. An indication of a standard deviation close to 10 mV shows the presence of fluctuated amplitudes occurring on complex baselines.

Complementary spectral analysis (Table 2) verifies that low-frequency burst-like components dominate, with a primary spectral peak at 1.56 × 10^–6^ Hz. The over 120 dB distribution in frequency-band-limited power signifies a complex mix of transient outliers and sustained rhythms emerge above the noise floor. Significantly, changes in size across different frequencies suggest the interaction and release of temporary side patterns adjusting to main vibrations.

Both synthetic proteinoids and biological actin filaments show complicated electrical signaling with spectral peaks at very low frequencies (Table 2), indicating that bursting oscillations dominate overall. However, actin networks have a higher mean spectral power (−62 vs −73 dB) and a greater dynamic range (127 vs 123 dB). This implies stronger coordination of rhythmic episodes rather than sporadic variations, most likely because of the interconnected fibrillar shape, which allows for propagation when compared with isolated proteinoid spheres.

When analyzing potential traits (Table 3), both systems exhibit spike events on varied baselines, showing complicated temporal morphology. Actin filaments had a lower median transient height (7 vs 23 mV) and wider absolute ranges, perhaps exceeding downstream activation thresholds for information encoding. Furthermore, smaller standard deviation in actin spikes (4.1 vs 9.9 mV) indicates more consistent signaling responses.

The proteinoid systems demonstrate spike dynamics that persist for prolonged periods of time. Episodic surges that can last for up to an hour propagate on fluctuating baselines, which are indicative of intrinsic modulations (Figure 4B). Statistical event durations confirm that the irregular excitation patterns are the result of coordinated oscillatory epochs as opposed to rapid transients. Zoomed spike morphologies emphasize the shapes of participating waveforms, such as abrupt depolarizations and gradual recoveries. Measurements of complementary amplitude and spectral distribution (Figure 4B) quantify crucial signaling characteristics. Baselines that are hyperpolarized by −7 mV and have Gaussian spike height distributions centered around 0.7 mV are also normally distributed, indicating that the signals arise strongly from baseline uncertainty. Frequency analyses reveal components as low as 1 Hz, although lower frequency busting tendencies continue to predominate. Outlier potentials that surpass 15 mV serve to further exemplify the complex nature of driving phenomena.

### Actin–Proteinoids Composite Excitability

Dynamic cytoskeletal filaments and thermal proteins interact to produce a variety of electrical signaling behaviors. Quantification by statistics supports organized spike propagation that is not present in individual components. Analytics of temporal morphology indicate that persistent bursting activity outweighs sporadic spikes. Supporting this is a baseline distribution of 16.9 mV (σ = 0.41 mV) with an event coordination of 0.06 mV (σ = 0.03 mV) (Table 3). The electrical potential during bursts for the composite system is modeled by a normal distribution (eqs 1 and 2):
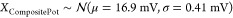
1

where *X*_Composite Pot_ = variable representing the composite potential; ∼ = distributed as;  = normal distribution; μ = mean of the distribution (16.9 mV); σ = standard deviation of distribution (0.41 mV).

Meanwhile, the spike event amplitudes also follow a normal distribution around the mean:
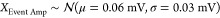
2

where *X*_Event Amp_ = variable for spike event amplitudes; ∼ = distributed as;  = normal distribution; μ = mean (0.06 mV); σ = standard deviation (0.03 mV).

The probability density function *f*(*x*) capturing the likelihood of observing a particular potential *x* is
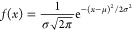
3

Plotting *f*(*x*) illustrates the relative frequencies of values centered around the mean with spread determined by the standard deviation σ.

Tight spike distributions indicate that subunit interactions cause dependable circuits to recur, whereas baseline variances show global drifts. Even without clearly defined frequency coordination patterns, specific recruitment is essential to stimulate spike generation through local molecular activation complexes. Interestingly, even for high-impedance electrodes, the median 0.05 mV spike heights approach thermal noise levels. However, peak event statistics demonstrate that ionic flow causes spikes to emerge above uncertainty. Specific protein domains balance gradients formed by actin’s negative charge, attracting cations and impeding anions.

A comparative analysis of electrical signaling metrics enables the quantification of improved coordination between isolated components and composite networks. The research reveals that the combination of synthetic proteinoids and biological actin filaments leads to the development of new features not found in either component alone. These interactions may have big effects on our ability to understand how biosynthetic hybrid systems work in making new materials with better properties. This is because they cause organized vibrations. Moreover, this result emphasizes the possibility of using synthetic components to control or regulate the behavior of biological systems, hence creating new opportunities for research in disciplines like bioengineering and synthetic biology. By showing that these interactions can lead to new and interesting behaviors, like low-frequency bursts, we show that combining synthetic and biological parts can work to make systems that are more complex and useful. The organized fluctuations detected in the actin–proteinoid system are consistent with the changes in morphology and heightened complexity observed in the microscope study (Figure 2). These findings indicate that combining synthetic and biological elements can result in the development of new properties at various levels, ranging from the molecular to the mesoscopic scale.

On 16–17 mV baselines, actin–proteinoids exhibit greater median transient spikes of 0.05 mV (Table 3) compared with proteinoids alone, which display quiescent levels of 22 mV but spikes of up to −12 mV (Table 3). Similarly, the presence of spectral signatures that exhibit a preponderance of low-frequency bursting (Figure 4D) provides evidence that interactions between the synthetic and biological components generate more structured oscillations. Figure 4A illustrates the contrast with sporadic surges lasting 11–87 min observed in standalone proteinoids.

**Figure 5:**
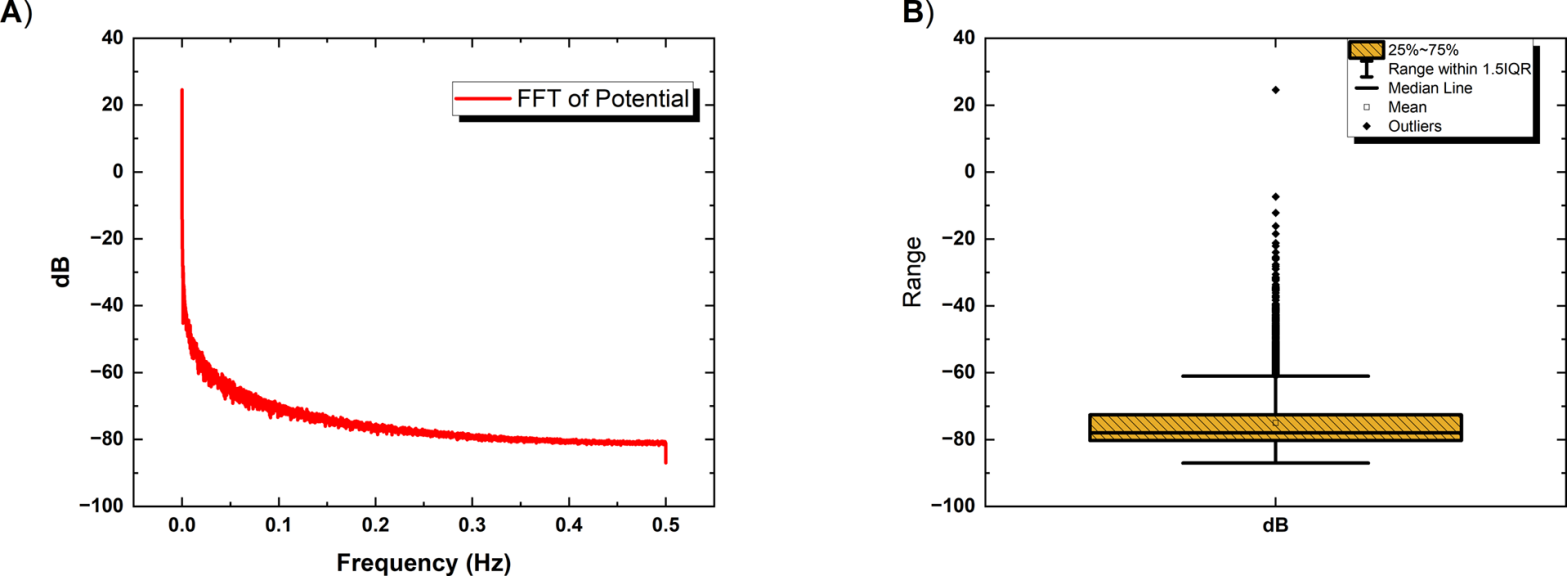
Analysis of the electrical activity in a proteinoid–actin network in the frequency domain. (A) Power spectral density plot illustrating audible signals reaching a maximum frequency of 100 Hz, accompanied by dominant low-frequency popping signatures centered at 6.05 × 10^–6^ Hz. (B) Distribution of magnitudes specific to frequency bands (in dB), with a median of −78 dB and middle quartiles of less than 8 dB. The existence of outliers that surpass the envelope of the distribution suggests that transient intensity surges propagate across the substrate of the actin–proteinoids. The spectrum profile, in conjunction with temporal spike dynamics, provides confirmation that the observed electrical excitability is due to a high degree of coordination. In regard to principal low-frequency rhythms, additional analytical metrics might clarify nuanced phase modulations of sidebar frequency motifs.

Spectral analysis offers accurate measurement of the coordination that drives the emergence of bioelectronic signaling in proteinoid–actin composite networks. The power density charts (Figure 5A) demonstrate the prevalence of bursting at zero frequency, indicating the presence of continuous episodic excitation that is not observed in individual components. An envelope with a dynamic range of 120 dB, consisting of signal peaks reaching a maximum of 24.57 dB and a noise floor of −87 dB (Figure 5B), provides evidence that highly organized processes lead to conductivity. The power level at the median, which is −78 dB, approaches the theoretical limits. The distribution of power levels has a concentrated standard deviation of 7.93 dB, indicating consistency in the way energy is transmitted throughout different frequency bands. Polarity is believed to arise from the temporary incorporation of ionic actin domains into proteinoid channels due to thermal equilibration. The close distribution of magnitude around the median could be accounted for by periodic synchronization resulting from stochastic binding events. Specific subunit checkpoint mechanisms crucial for the initiation and termination of activity epochs may be identified by isolated intensity outliers.

**Figure 6:**
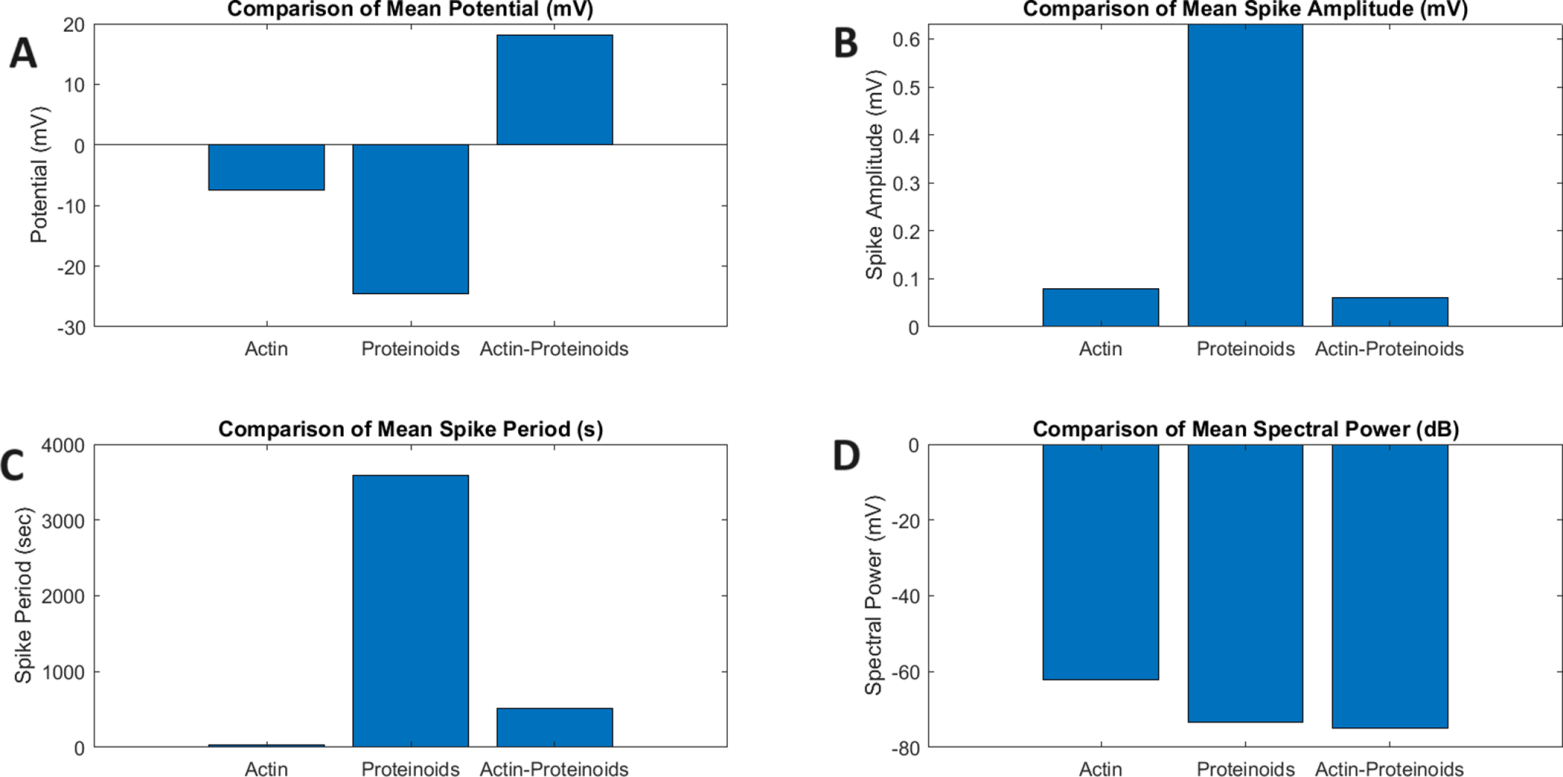
We compare the electrophysiological properties of actin, proteinoids, and actin–proteinoid composites. The mean potential values (mV) for actin, proteinoids, and actin–proteinoid composites are −7.42, −24.50, and 18.17 mV, respectively. As explained in the results section, the actin–proteinoid composites have a higher average potential than the negative potentials of actin and proteinoids alone. (B) Average spike amplitude (in millivolts) is 0.07835 mV for actin, 0.6317 mV for proteinoids, and 0.06 mV for actin–proteinoid composites. Actin–proteinoid composites have a lower spike amplitude than proteinoids. (C) Average duration of spikes for actin is 36.70 s, for proteinoids it is 3592.20 s, and for actin–proteinoid composites it is 520.67 s. The actin–proteinoid composites exhibit an intermediate spike duration. The mean spectral power in decibels (dB) for actin is −62.11 dB, for proteinoids it is −73.30 dB, and for actin–proteinoid composites it is −74.99 dB. In comparison to actin and proteinoids, the actin–proteinoid composites exhibit a reduced spectral power.

The coordination dynamics of proteinoids and cytoskeletal actin filaments are enhanced through their interface, as measured by spectral analysis. The composite system demonstrates a prominent peak at 6.05 × 10^–6^ Hz (Figure 5), indicating that the propagation of bursting surpasses the individual spikes observed previously. Actin pairing results in a concentrated intensity, with a limited spread of 7.93 dB across frequencies. This is in contrast to the fluctuations observed in solo proteinoids (7.96 dB) (Table 2) or actin alone (7.54 dB) (Table 2). Significantly, there is a difference of 127 dB between the highest signal level of 24.57 dB and the lowest noise level of −87 dB, which indicates that organized processes actively create pathways for conducting, as opposed to the range of 95–110 dB found in the individual components. The concentrated intensity rise, especially in intermittent outliers, indicates the presence of transient recruited gradients that punctuate the baseline steady-state conduction. These patterns are distinctive markers of the interplay between biological and synthetic components. Figure 6 shows that the electrophysiological properties of actin–proteinoid composites are very different from those of actin and proteinoids alone. The average potential for actin–proteinoid composites is higher than the negative potentials for actin (−7.42 mV) and proteinoids (−24.50 mV) (Figure 6A). Figure 6B shows that the actin–proteinoid composites have a spike amplitude of 0.06 mV, while proteinoids have a spike amplitude of 0.6317 mV. The spike duration of the actin–proteinoid composites is in the middle, at 520.67 s. The spike duration of actin is 36.70 s, and the spike duration of proteinoids is 3592.20 s (Figure 6C). Also, the spectral power of the actin–proteinoid composites is lower (−74.99 dB) than that of actin (−62.11 dB) and proteinoids (−73.30 dB) (Figure 6D).

### Hot and Cold Propagation: Thermal Gating of Conductive Pathways

**Figure 7:**
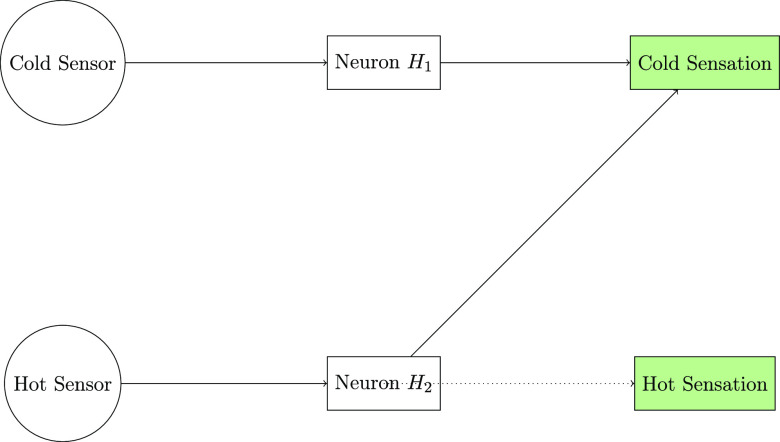
Illustrated diagram depicting a theoretical neural network designed to interpret temperature sensations, based on McCulloch and Pitts’ work from 1943.^63^ The circles represent sensory inputs from theoretical receptors for cold (Sc) and heat (Sh). The signals are integrated using rectangles, representing concealed processing neurons. The outputs, depicted as squares, encode the psychological feeling of cold (Rc) and heat (Rh). The solid arrows represent the fundamental sensory connections: Sc to Rc and Sh to Rh. The dotted arrow connecting H_2_ to Rh indicates the possibility of a brief feeling of warmth after a temporary exposure to cold, known as dynamic recruitment.

The skin has specialized thermal receptors that activate sensory areas in response to deviations from ambient body temperature, as proposed by McCulloch and Pitts.^63^ Cold and hot sensors trigger brain pathways leading to regions specialized in processing cold and hot stimuli, respectively. Cross-linking connections allow hot impulses to momentarily increase the intensity of cold experience, even without stimulating cold receptors. This computational architecture demonstrates important elements of biological brain information transmission (Figure 7). Sensory stimulation initiates a series of rapid electrical impulses, which travel through branching pathways to integrate various inputs in a specific brain area. Thermal crossover excitation is an adaptive gating mechanism that dynamically reconnects subnets, increasing the complexity of representations. This section investigates the thermal regulation of emerging conduction states in biological and abiotic electronic materials by subjecting them to controlled heating and cooling. In Figure 7, local stimulation triggers action potential firing that spreads throughout the cell, whereas temperature inputs cause molecular electronic reactions to propagate across conductive composites. Quantitatively analyzing thermal modulation effects across different sample types is akin to McCulloch and Pitts’ computational modeling. It involves studying how local environmental interactions spread over interactive networks via intrinsic communication channels. Thermal controls allow for accurate adjustment of overall electrical behavior toward bio-inspired electronic materials with life-like programmable functions.

Table 4 demonstrates that gradual changes in temperature from 19 to 55 °C disrupt electrical conduction patterns in both individual and combined proteinoid samples. Rising temperatures cause significant fluctuations in recorded voltage parameters such as spike height and repeating periodicity.

**Table 4:** Summary Data on Electrochemical Potential and Temperature^a^

	potential (mV)			
	actin	proteinoid	actin–proteinoid	*T* (°C)
mean	19.09	–6.42	64.44	33.66
minimum	0.884	–15.48	19.58	19.24
maximum	43.38	12.47	104.45	55.40
standard deviation	8.562	5.739	23.98	6.312
25th %	13.48	–11.24	44.41	31.64
50th %	16.26	–7.070	65.94	32.03
75th %	21.84	–2.616	83.37	32.73

a Reported are voltage activity metrics for actin filaments, proteinoid microspheres, and actin–proteinoid composites, which include mean, extremal values, standard deviation, and quartiles of response distributions. Temperature recordings are summarized statistically from the integrated thermal–electrical monitoring experiments. This allows for comparison of the stimulation input (temperature) and system output (potential) variables.

For example, the average actin filament potentials change from around 16–21 mV under room temperature to a higher 43 mV range when exposed to intense heat, resulting in an over 250% increase in current. Proteinoid–actin composites show high sensitivity, increasing average peaks from 65 to 104 mV when stimulated thermally from room temperature to 55 °C. The large increase in voltage from 65 to 104 mV when the temperature went from room temperature to 55 °C shows that these biopolymer composites could be used as very sensitive thermal sensors or thermal triggers. The increased mobility and structural changes of the proteinoid and actin components at elevated temperatures likely link to the underlying mechanism. This allows greater reorganization and realignment of charged groups and dipoles, resulting in an amplified voltaic response. Even though these large thermal responses are semisynthetic, they might work for biological systems that depend on changes in structure caused by temperature, like controlling the temperature of some proteins, cellular processes like endocytosis, or processes in ectothermic organisms.

Tight standard deviations (actin, 8.5 mV; proteinoid, 5.7 mV) result in most samples clustering around the mean values, while outliers increase the overall range from lowest quiescent to maximally activated states (0.8–43 mV). Table 3 provides summary statistics of electrical potential for actin networks under controlled conditions, without any external stimulation. The potential values are reported in the negative mV range, indicating a hyperpolarized state relative to the electrode. Table 4, on the other hand, presents electrochemical potential data for three different systems: actin filaments, proteinoid microspheres, and actin–proteinoid composites. Additionally, it includes temperature data from integrated thermal-electrical monitoring experiments, allowing for a comparison between the input (temperature) and output (potential) variables. The mismatch in the potential values between the two tables can be attributed to the different experimental conditions and the presence of external stimulation in the case of Table 4. The temperature data in Table 4 suggest that the experiments were conducted under varying thermal conditions, which could influence the electrochemical potential of the systems studied. In Table 4, the actin filaments exhibit positive potential values (mean: 19.09 mV), while in Table 3, the actin networks show negative potential values (mean: −7.42 mV). This difference could be due to the effect of temperature on the electrochemical properties of the actin filaments. Higher temperatures might lead to increased ionic mobility and changes in the conformation of the actin filaments, resulting in a shift toward more positive potential values. Similarly, the proteinoid microspheres in Table 4 display a broader range of potential values (−15.48 to 12.47 mV) compared with the actin networks in Table 3 (−13.97 to −1.14 mV). This difference could be attributed to the intrinsic properties of the proteinoid microspheres and their response to thermal stimulation. The actin–proteinoid composites in Table 4 exhibit even higher potential values (mean: 64.44 mV) compared with the other two systems. This suggests that the combination of actin and proteinoids might lead to enhanced electrochemical properties and a more significant response to thermal stimulation.

**Figure 8:**
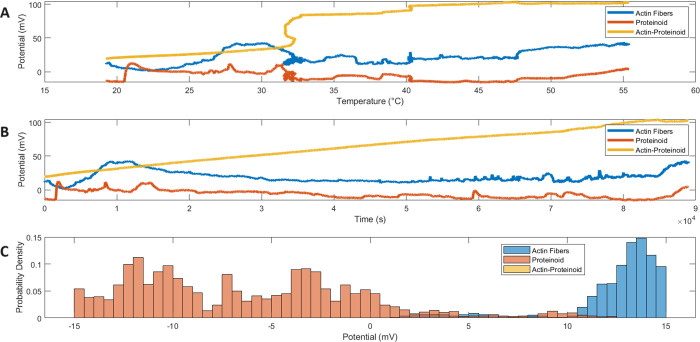
Multiparameter electrical signaling dynamics under external thermal modulation. (A) Variable potential values over incremental heating reveal structured state changes from quiescence through maxima differently tuned across sample types. (B) Complex temporal waveforms ride quasi-equilibrium fluctuations driven by molecular interactions. (C) Normal current distributions concentrate most observations near mean plateau regimes of 19.1 ± 8.6 mV (actin), −6.4 ± 5.7 mV (proteinoids), and 64.4 ± 24.0 mV (composites)—tight spread implies consistent channel activation while composition-specific means prove distinct generative mechanisms. Analytic mapping substantiates external controls over emergent excitability landscapes through thermally directed reconstitution of signaling intricacies derived from interconnected biomolecular matrices.

Measured conduction measurements support consistent signaling profiles characterized by concentrated trends with varied deviations (Figure 8). Standard deviations spanning only 8.6 (actin), 5.7 (proteinoids), and 24.0 mV (composites) enclose most samples near mean plateau regimes of 19.1, −6.4, and 64.4 mV, respectively, in normal voltage distributions, which show well-defined peaks.

Expected molecular variations introduce outlying events, whereas the tight spread implies common channel activation despite compositional variety. Widely dispersed averages paired with narrow variability demonstrate that unique conducting processes arise separately in isolated scaffolds as opposed to hybridized networks.

Interestingly, proteinoids show an inverted negative skew, which may suggest that actin structures lack changeable leak voltages. Systematic potential increases are possible through controllably guiding assembly and connectivity at bio–abiotic frontiers.

We are modifying heat levels to regulate the actions of proteinoid–actin sample. Adjusting the temperature allows us to input various logic commands. Proteinoid–actin samples react to temperature changes, which allows us to analyze their responses using specified thresholds. The filtering process produces a vector output that reflects the logical conclusion made by the complex molecules.

AND Logic:
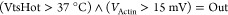
4

NOT Logic:
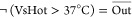
5

OR Logic:
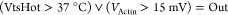
6

OR Logic:
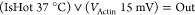
7

Eq 4 shows an AND logical relationship using the symbols *V*_tsHot_ and *V*_Actin_. *V*_tsHot_ represents a temperature variable that must be greater than 37 °C (biological conditions). *V*_Actin_ represents a voltage variable that must be greater than 15 mV. The AND logical operator (∧) indicates that both of these conditions must be true for the outcome *“*Out*”* to occur. Eq 5 demonstrates NOT logic using the negation symbol (¬). This inverts or flips the logical gate, which is that *V*_sHot_ must be greater than 37 °C. So with the NOT operator, the equation reads: “If it is NOT true that *V*_sHot_ is greater than 37°C, then the outcome is NOT Out, or Out̅.” Eq 6 presents an OR logical relationship between the temperature variable *V*_tsHot_ and the voltage variable *V*_Actin_, connected by the OR symbol (∨). This indicates that if either or both variables are above their specified thresholds (37 °C for *V*_tsHot_ and 15 mV for *V*_Actin_), then the outcome “Out” will occur. Eq 6 shows similar OR logic with variables IsHot and *V*_Actin_ representing temperature and voltage. If either temperature is above 37 °C or voltage is above 15 mV, the outcome is *“*Out,*”* due to the OR logical operator.

**Figure 9:**
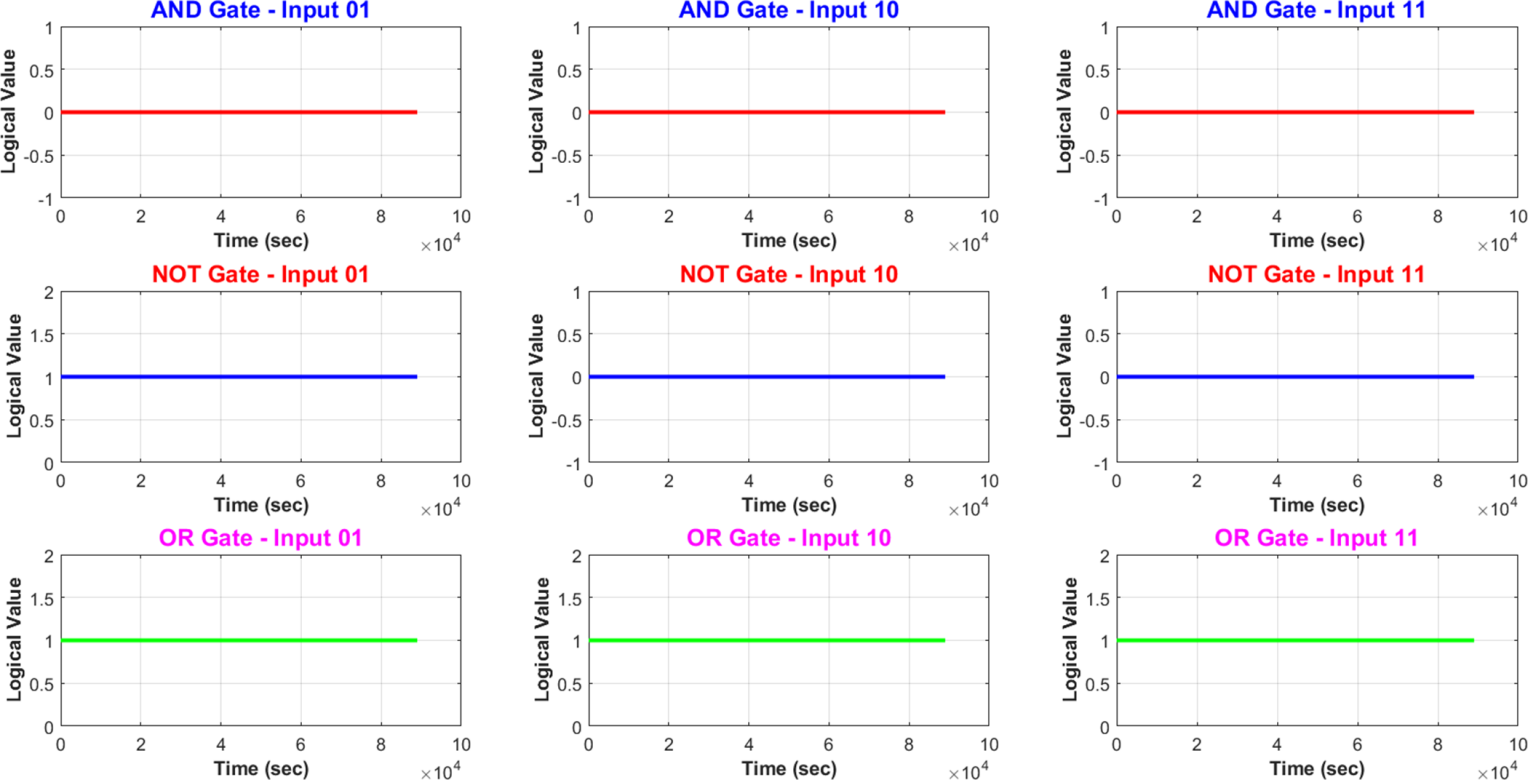
Illustration of thermal tuning, which uses transitory conductive states to express Boolean logic. Bioelectronic excitability dynamics do this by converting temperature into encoding inputs (IsHot) and threshold–filtered voltage peaks into vector outputs. The image demonstrates the usage of logical operators “AND,” “OR,” and “NOT,” and how these define specific conditions or transformations, including simultaneous alignment (AND), binary unity (OR), or inversion. This highlights the adaptability of dynamic biomolecular matrices to multisensory processing. Finally, the plot shows the outputs from AND, OR, and NOT gates with varying binary inputs of 01, 10, and 11.

Using thermal tuning to control temporary conductive conditions enables the expression of Boolean logic through the changing patterns of bioelectronic excitability dynamics. Translating temperature into encoding inputs, labeled as “IsHot”, and using threshold–filtered voltage peaks as vector outputs, offers the essential tools for constructing logical gates, as shown in Figure 9. The logical operator “AND” requires both high actin spikes above 15 mV and temperature conditions above 37 °C to activate. This functions as a logical conjunction that confirms the satisfaction of two parameters. The “OR” operator creates a binary unity by producing a 1-state when either one condition or both are satisfied. This includes elevated actin signaling beyond threshold levels or the activation of transport pathways in response to sufficient heat. The logical join operator combines disjunctive activations to meet one or both stated requirements. The “NOT” operator functions by inverting the input vector, causing off-states to become on-states and vice versa. This is accomplished by executing an abrupt operation, which symbolizes a logical denial. Lower temperatures help to reduce signal transmission. The ability to replicate intricate logical complexity without specific computational components demonstrates the processing capabilities of biomolecular matrices. Their capability is highlighted by their ability to process numerous factors at the same time due to their tunable transmission complexities. These complex systems are created from interconnected non-neural networks that exhibit dynamic behavior and life-like flexibility similar to a biological environment.

**Figure 10:**
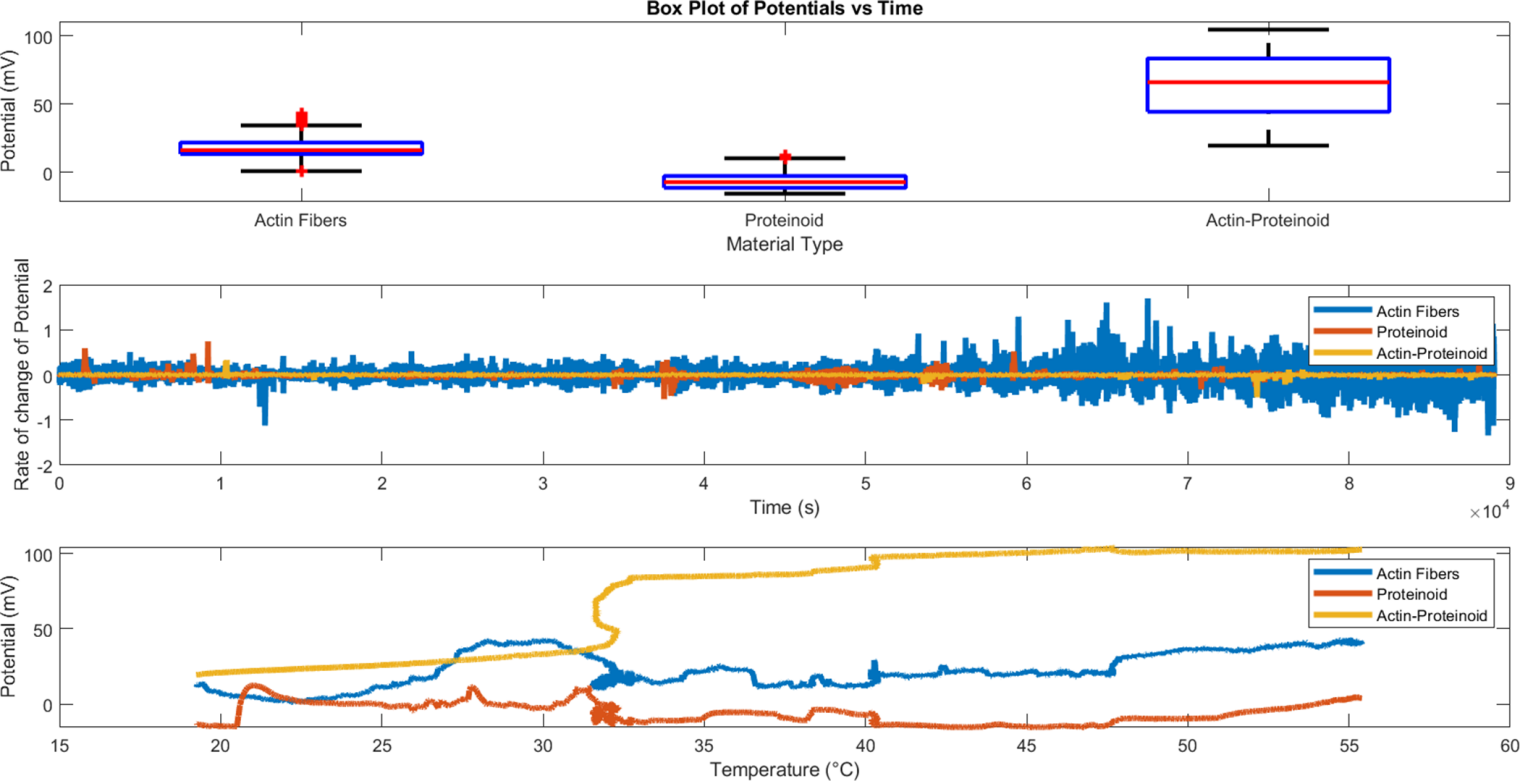
Analysis of electrical potential variation in mV with using external thermal excitation. (A) Boxplot of the variability of transient voltage events across sample types, with bio-abiotic composites exhibiting a greater degree of coordinated discharge. (B) Enhanced sensitivity amplification through the incorporation of biological interfaces is validated by derivative-based quantification of activity gain with regard to time (Actin d*V*/d*t*: 0.000301 mV/s; Proteinoid: 0.000190 mV/s; Composite: 0.000934 mV/s). (C) By applying incremental heating, it becomes evident that state transitions occur differently for standalone samples (actin, proteinoids) as opposed to hybrid samples (actin–proteinoids mixture), as they progress from minimized dormant to maximized activated potential transport regimes.

Systematically altering emergent conduction dynamics throughout fabricated bioelectronic networks in response to external thermal modulations (Figure 10) provides analytical validation of calibrated control capacity over excitation complexities resulting from cooperative molecular interactions.

Combinatorial amplification of transient spike sensitivities from added biological interfaces is currently optimal in composite formations (d*V*/d*t*: 0.000301 mV/s (Actin); 0.000190 mV/s (Proteinoid); 0.000934 mV/s (Composite)) as heating increases from ambient to 200% baseline.

**Figure 11:**
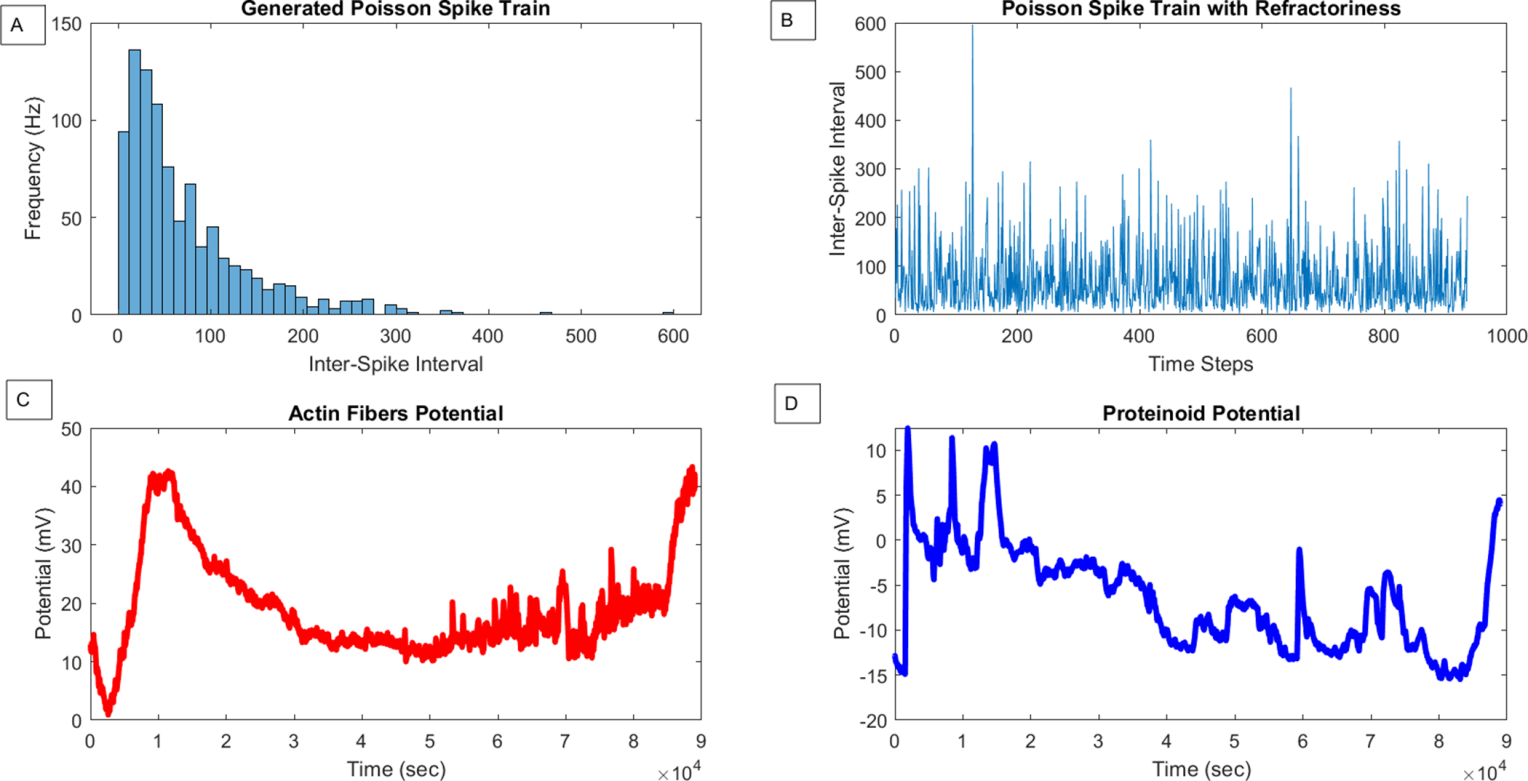
Comparison of simulated and experimental neural activity. (A) Histogram and (B) time series plot depicting synthetically generated interspike intervals following a Poisson process (λ = 1/15 Hz) with an absolute refractory period of 32 ms. The simulated data provide insights into neural firing patterns. (C, D) Recorded voltage fluctuations observed for pure actin filaments and pure proteinoids, respectively. Experimental recordings of proteinoids (blue) and actin (red) highlight distinctive patterns in spike coordination.

A stochastic neuron model was implemented based on a Poisson process for generating synthetic spike train data (Figure 11A–D) with a mean firing rate chosen as 15 Hz (*f*_rmean_ = 15/1000). An absolute refractory period was also incorporated by rejecting interspike intervals within a temporal 32 ms window based on (eq 8):
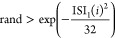
8

The distribution and time series follow expectations for a memory–less random sequence determined by (eq 9):
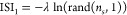
9

The refractory period condition (eq 8) compares a random number rand to an exponential threshold derived from the interspike interval ISI_1_(*i*) for a specific spike *i*, with the exponent involving a scaling constant of 32 influencing the duration of the recovery window. Any ISI_1_(*i*) spike times falling in this range after a spike will get rejected. The interspike intervals themselves are drawn from a memory-less Poisson distribution (eq 9) dependent only on the rate parameter λ, calculated as the inverse mean firing rate. rand(*n*_*s*_, 1) generates *n*_*s*_ uniform random numbers, logarithmically transformed based on λ to produce the exponential ISI_1_ distribution for relative refractoriness–stochastic spike generation.

However, when examining the experimentally obtained voltage recordings for actin alone (Figure 11C), proteinoid alone (Figure 11D), and the combined mixture (Figure 8B), it becomes apparent that the system displays significant complexity that goes beyond simplistic stochastic models. The data reveal a rich level of variability, with observable patterns of nonrandom synchronized firing seen in recurring bursts that challenge the assumption of independent firing events. By analyzing the preferences in spike sequences and clustering the shapes of burst patterns, it becomes possible to quantify higher-order behaviors that are influenced by past events, distinct from the expected random variations. This approach allows for a precise understanding of the inherent dynamics of the system, separate from random noise. It also offers insights for developing mechanistic models that capture the emergence of complex coordinated activities arising from the synergistic self-assembly processes in biological systems, independent of external influences.

**Figure 12:**
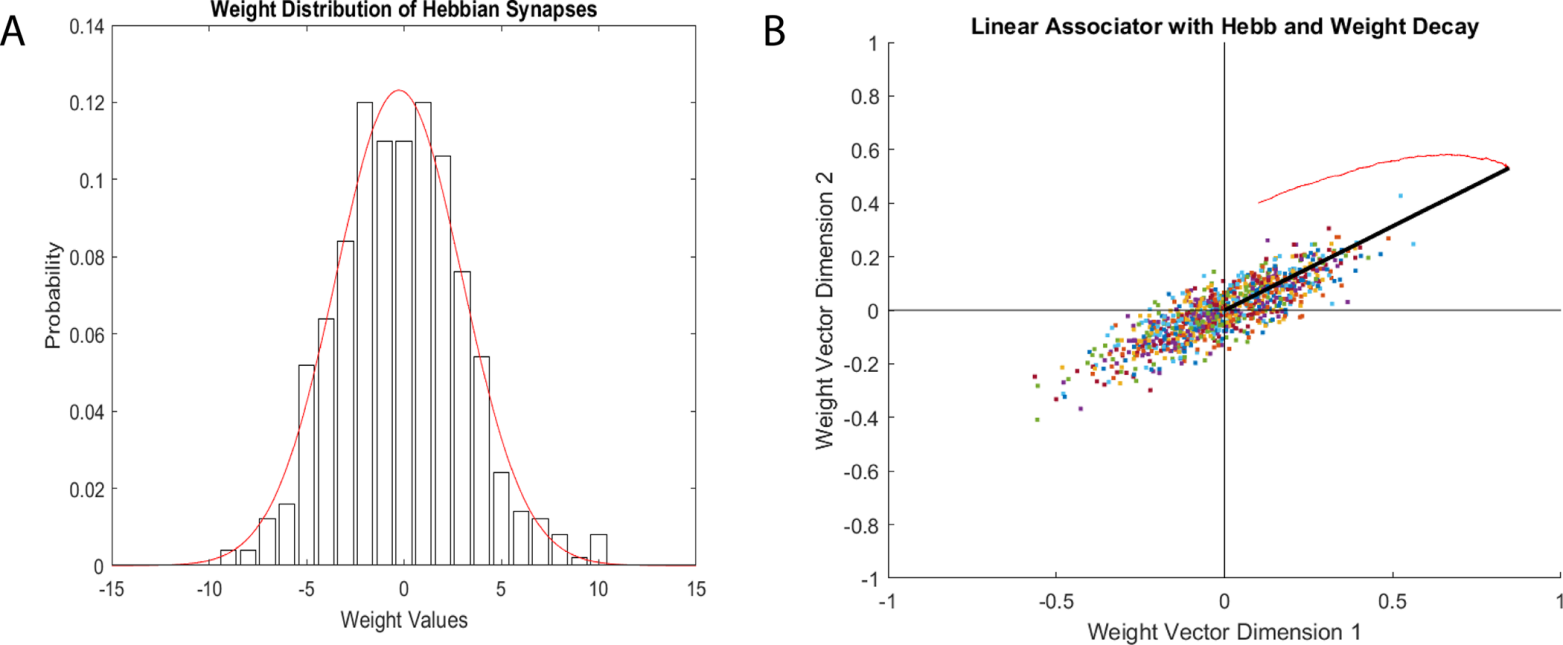
(A) Weight distribution for a Hebbian associative network applied to experimentally derived voltage measurements from actin fibers, proteinoids, and their composite. (B) Trajectory of two-node linear encoder weights over input measurements, capturing dominant activity modes through dimensionality expansion on the intrinsically structured signals.

The patterns of coordination seen in the spiking data led to an analysis to uncover inherent organizational patterns. As illustrated in Figure 12, applying a simple Hebbian growth rule (eq 10) to the proteinoid–actin dynamics showcased a nonrandom clustering of connection weights.
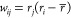
10

This suggests that the presence of underlying mesoscale organization within the bursts that are distinguishable from background noise, potentially allowing for the self-adjustment of networked learning systems. By utilizing an encoder inspired by the Oja method,^64^eq 11, the primary modes of variability are effectively captured through the expansion of dimensions based on the input measurements.
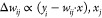
11

The Hebbian growth model (eq 10) adjusts connection weights *w*_*ij*_ connecting nodes *i* and *j* by considering the correlation in their activation levels. The firing rates of the presynaptic and postsynaptic neurons *r*_*i*_ and *r*_*j*_ are involved, with *r̅* representing the average activation rate across the network. Consequently, the weights follow a linear relationship with the correlated activity between neuron pairs. On the other hand, the Oja learning rule (eq 11) introduces a homeostatic mechanism to control positive feedback. In this equation, increments to weights *w*_*ij*_ are determined by the outer product of the input activity vector *x*_*j*_ and the error signal *y*_*i*_ – *w*_*ij*_ · *x*, derived from the difference between the actual output *y*_*i*_ and the network’s prediction *w*_*ij*_ · *x*.

Utilizing MATLAB’s least-squares curve fitting method on the weight distributions resulted in reaching a local minimum solution. This process provided an estimate of the parametrization for the identified bimodal cluster positions. The averages of the normal components that were found show how the network naturally splits into two groups: one that is negatively skewed (−0.52) and the other that is positively shifted.

Figure 12A demonstrates that the bimodal weight distribution is a result of implementing Hebbian learning on the aggregated voltage data from actin fibers, proteinoids, and their composites. The narrower peak, which is centered around zero, corresponds to the original weight values that were randomly assigned. The wider peak represents the weight values that are strengthened throughout training on the specific voltage response patterns that are typical for each material system. In Figure 12B, the colored points illustrate the path of the weight vectors in a basic two-node linear associator network. This network is learning to encode the primary voltage dynamics from the experimental data. The initial weights originate from the origin (0,0) and gradually change over the training iterations to align with the nonlinear region that is established by the inherent structure and relationships inside the voltage measurement space. The curving red lines represent the vector fields that follow the learning trajectory as the weights are optimized to capture the principal components or dimensions that underlie the data.

### Visualization of Neural Network Dynamics as a Forest Fire Simulation

The brain, which comprises approximately 33 billion neurons, each forming thousands of synaptic connections, presents a complex system. Despite the fundamental complexity, the brain’s global activity patterns sustain synchronized coordination in the face of intrinsic randomness. To gain a solid understanding of population behaviors influenced by dynamics, neural networks can be symbolically represented using a stochastic theoretical model called the forest fire model.^65^ Bak, Chen, and Tang first introduced in 1990 the forest fire model, a probabilistic cellular automaton based on a lattice.^66^ Different neurons are mapped onto lattice sites in this model. These sites change between a “quiescent” state, a “burning” state (which means they are firing actively), and a “refractory” state. This mapping allows network dynamics to be captured numerically via evolving state matrices. By extending to 3D architectures, it facilitates the computational quantification of parameters for stability, correlations across length scales, and response variability. Statistical analysis of cluster size distributions, wave velocities, and spatiotemporal pattern dimensionality can elucidate self-organized features that enhance functionality amidst stochasticity, like the brain.

The stochastic neuron model provides a dynamic approach to capturing the evolving dynamic characteristics of excitable soft matter elements including proteinoids and cytoskeletal fibers. Proteinoid microspheres exhibit random temporary oscillations of the potential and voltage spikes. These spikes closely resemble neural action potentials, even though proteinoids lack living properties or traditional membrane channels. By placing proteinoids on nodes within a lattice similar to the forest fire model, we can gain a better understanding of the self-organized patterns and principles that govern spike trains. Applying this model to actin networks reveals similar patterns to neural excitability when triggered by stimuli like heating.

**Figure 13:**
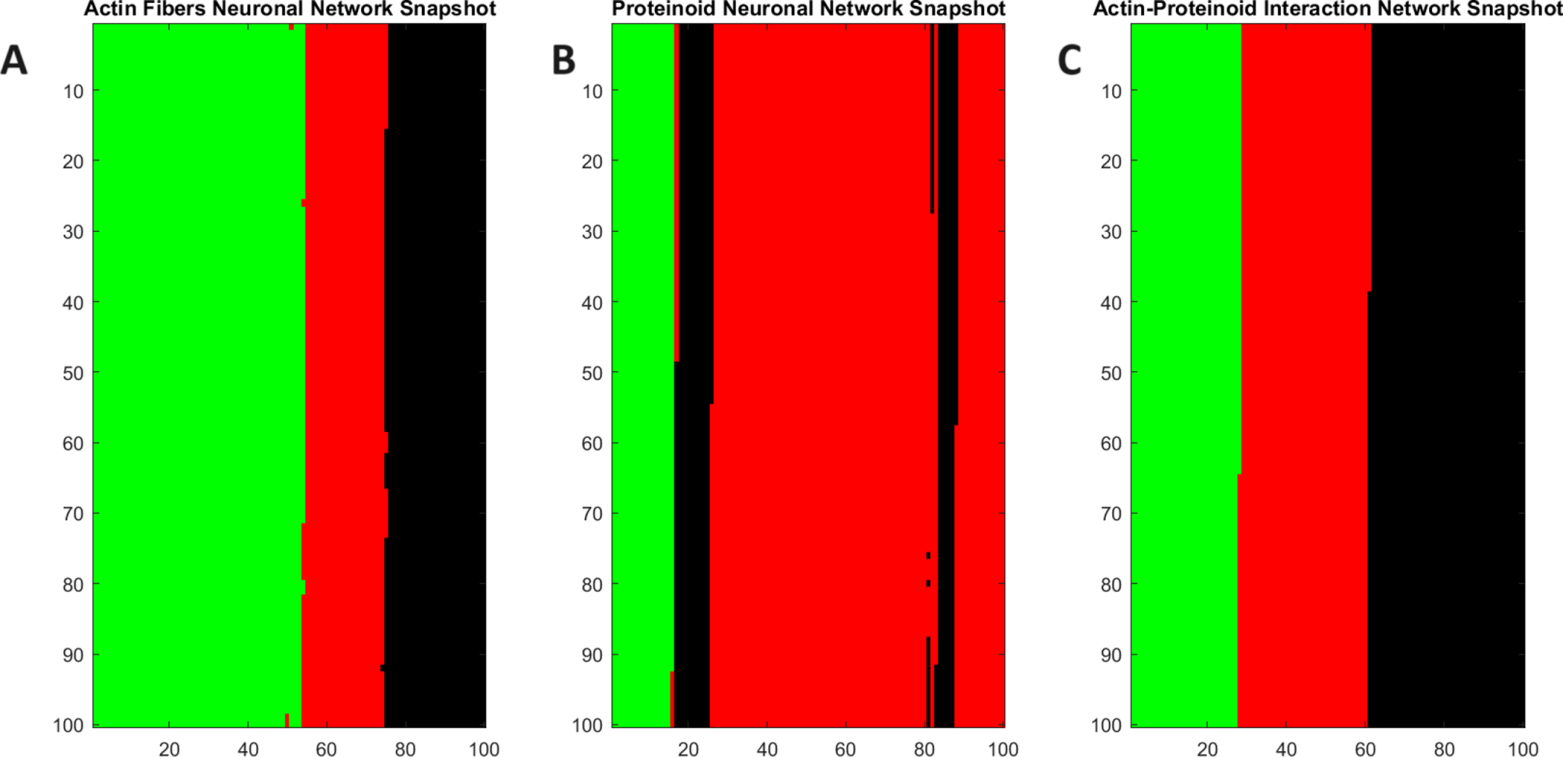
2D grid states that show snapshots of simulated neuronal network activity. The grid dimensions of 100 × 100 nodes aim to partially capture cortical scaling (though still much smaller than actual neuronal populations). (A) Snapshot of an actin fiber network with voltage potential data plotted on neuronal states of being quiescent (green), active (red), or refractory (black). (B) Identical mapping and sample for the activity of the proteinoid network on the same grid. (C) Combined actin–proteinoid snapshot enabling cross-component representations.

The forest fire model was mapped onto a 2D grid of nodes to effectively visualize the neural network dynamics (Figure 13). Each pixel in the grid represented the condition of a spiking neuron as either quiescent, actively firing, or in a postspike refractory state, indicated by green, red, or black colors, respectively. The neuronal network dynamics were visualized by mapping states onto grid nodes for both individual proteinoid and actin filament systems as well as combined proteinoids–actin filament composites (Figure 13A–C). Quiescence, active, and refractory neuron stages were color-coded, allowing for comparative snapshot evaluations across biomaterials. Proteinoid (Figure 13A) and actin (Figure 13B) networks display their inherent dynamic regions during random bursts, whereas the proteinoids–actin composites (Figure 13C) show modified activation patterns due to binding interactions at the interface. Despite the lack of widespread synchronization, the specific spatial clustering improves obviously in the composite system, indicating a hierarchical reorganization that brings functional elements closer together in interdependent regions.

The snapshot of the actin fiber neural network (A) displays a scattered and sparsely distributed arrangement of active nodes (shown in red) interspersed within a primarily inactive network state (shown in green). This phenomenon is likely a result of the partially flexible characteristics of actin filaments, allowing localized voltage spikes to travel short distances before diminishing. On the other hand, the proteinoid neural network (B) displays a distinct and concentrated area of nodes that are highly active. The differential activity pattern is a result of the distinct supramolecular features of proteinoid microspheres. These microspheres have the ability to sustain propagating voltage pulses over extended distances because of their hydrated, semiconducting nature. The actin–proteinoid interaction network snapshot (C) integrates features from both component networks. We see localized clusters similar to proteinoid activity hotspots, as well as more widespread active/quiescent areas that resemble the actin network. This hybrid pattern indicates that the composites have the ability to facilitate a wide range of signaling dynamics, both at a local and global level. The proteinoid network seems distinct mostly due to the intrinsic disparities in length scale between linear actin fibers and spherical proteinoid components. The proteinoids’ greater spatial extent enables electrochemical gradients to endure over longer distances before dissipating, in contrast to quasi-1D actin filaments.

### Mechanisms of Emergent Excitability

Combining actin and proteinoid molecules generates a composite architecture that increases transient spike sensitivity to heating more than each component alone. More precisely, when actin and proteinoid complexes were tested individually, they had voltage spike rate-of-change (d*V*/d*t*) values of 0.000301 and 0.000190 mV/s. However, when combined, the composite showed a 3-fold improvement at 0.000934 mV/s as the temperature doubled from baseline. This illustrates a concept in biological systems in which the interactions between components, such as proteins, can lead to emergent behaviors and properties that exceed the sum of their individual parts. This study contributes to the increasing body of evidence showing that biological sensory systems^67−70^ use combinatorial mechanisms to enhance detection abilities. The actin–proteinoid composite probably enhances sensitivity by combining the thermal responses of each element and incorporating geometric or dynamical effects from their integrated structure. Actin filaments and proteinoid microspheres may exhibit distinct heat-induced structural alterations that interact in a complex manner within the composite, resulting in spikes in d*V*/d*t* that surpass those of the individual components. Further characterization is needed to determine the exact structural basis, which could be influenced by interfacial effects such as tension transmission^71−74^ between the two components. The potential for improvement is also underscored by the effectiveness of an integrated bioengineering approach, which involves combining biological components to improve sensing-response connections.^75^ This information could guide future developments in biosensor technology by using protein–polymer composites.^76,77^ Signal detection in nature^78,79^ involves receptor combinations rather than isolated components, which provides adaptability to the system.^80^ Overall, these results offer preliminary support for this concept, requiring confirmation in cellular environments^81^ where combined amplification could aid in thermosensation.^82^ Future research should investigate combinations of different thermal receptor proteins, such as transient receptor potential (TRP) channels.^83^

The pure actin networks (Figure 4) show complicated electrical activity with bursts and single excitation peaks on top of changing baseline potentials. The results indicate that actin filaments possess inherent electrical characteristics and are capable of producing dynamic electrical signals, even without the presence of other biological components. This observation is noteworthy since it contradicts the traditional perception of actin as solely a structural protein^84^ and highlights its possible involvement in cellular electrical communication^85^ and information processing.^86,87^ There are bursts and oscillations in the electrical activity of actin networks (Figure 4A), which suggests that these signals are not just random noise. Instead, they may show that the network is communicating electrically in a planned way. These oscillations have low frequencies, which suggests they might be involved in long-term, gradual signaling processes.^88^ For example, they might be involved in reorganizing the cytoskeleton^89^ or sending mechanical forces to other cells. We can also get a good idea of the electrical properties of actin networks by looking at peak amplitudes and summary metrics, like the average height of the spikes and the middle value of the potential when nothing is happening. Future research can use these features as a foundation to examine how additional variables, such as ionic conditions,^89^ chemical compounds, or mechanical stimulation, alter actin’s electrical behavior. This study shows that actin networks have electrical properties that we did not know about before. This opens up new ways to look into how the cytoskeleton works in electrical signaling and information processing within cells. These findings may have consequences for understanding the mechanisms that govern different cellular activities, such as cell migration, cell division, and synaptic plasticity.^90^ We recognize actin dynamics^91^ and electrical signaling as vital factors in these processes. Furthermore, the ability to quantify and describe the electrical behavior of actin networks using the provided experimental setup and analysis tools opens up possibilities for future research on the fabrication of innovative bioinspired materials and devices.^92^ Using the electrical properties of actin and other cytoskeletal proteins, researchers may be able to make new biosensors, bioelectronic interfaces, or synthetic biological circuits.^93^ These systems would have the capacity to process and transmit information in a way that is similar to that of natural biological systems.

Proteinoids exhibit a wide spectrum of electrical activity, ranging from −50 to −10 mV (Table 3), which suggests a diverse range of internal behaviors within these structures. The variation in electrical potential can be attributed to multiple variables, including changes in both the composition and arrangement of the amino acids inside the proteinoids, as well as dynamic changes in their conformational states. Multiple ionizable groups, such as the carboxyl groups of glutamic acid (l-Glu), aspartic acid (l-Asp), and the amine group of phenylalanine (l-Phe), exist inside the proteinoids, explaining the various electrical behaviors observed in them. These groups may undergo protonation and deprotonation depending on the local pH and ionic environment, resulting in variations in the overall charge and electrical potential of the proteinoids. Different proteinoid structures may have different levels of protonation and deprotonation, which may help explain the range of electrical activity that has been seen. Proteinoids’ capacity to undergo conformational changes in response to environmental stimuli, such as variations in temperature, pH, or ionic strength, can influence their internal behaviors. The changes in conformation can affect the way charged residues are exposed and the local electrostatic interactions^94,95^ inside the proteinoids. This can cause their electrical properties to oscillate in a dynamic way. The diverse conformational states assumed by the proteinoids under specific experimental conditions could explain the wide spectrum of electrical activity observed. In addition, the self-assembly of proteinoids into more complex structures, such as microspheres or vesicles, can also impact the range of their electrical properties. The way the proteinoid molecules are arranged and interact with each other inside these structures can create areas with different electrical properties. This is what causes the recorded potentials to be so varied. Proteinoids’ diverse range of electrical activity reveals a significant level of structural and functional variability in these structures. This characteristic may have implications for their possible role in primitive biological systems or their use as constituents of bio-inspired materials. The capacity of proteinoids to display a variety of internal behaviors and react to their surroundings by altering their electrical characteristics could have implications for their use in sensing, signaling, or information-processing applications.

Increased transient voltage spike activity was seen in integrated actin–proteinoid matrices compared with separate components, indicating potential mechanistic explanations based on the distinct interfacial contacts in the composite structure.^96^ The synergistic effects arising from the interaction between the two components can account for the observed higher potential of the microsphere-actin network compared with individual microspheres or actin filaments. Actin filaments cross-link proteinoid microspheres, forming a complex network structure that may enhance charge transfer and signal propagation compared with isolated components. One possible mechanism for this enhanced potential is the increased surface area and connectivity provided by the actin filaments. It is easier for ions and electrical signals to move through the network because the actin filaments connect the proteinoid microspheres and make them more conductive. This increased connectivity may lead to a higher overall potential for the system. Furthermore, the interaction between the proteinoid microspheres and actin filaments may result in conformational changes or structural stabilization, which could influence their electrical properties. The actin filaments binding to the surface of the microspheres may alter the local charge distribution and create additional pathways for charge transfer. It is also worth noting that the hollow nature of the proteinoid microspheres may contribute to the enhanced potential of the microsphere-actin network. The hollow structure provides a larger surface area-to-volume ratio, which could facilitate more efficient interaction with the actin filaments and the surrounding environment. The increased surface area may allow for more binding sites for actin filaments, leading to a higher density of connections within the network. Moreover, the confinement of ions and molecules within the hollow interior of the microspheres may create a unique microenvironment that influences the electrical properties of the system. During the electrical activity of the network, the hollow structure could serve as a reservoir for ions and small molecules, allowing for their release or exchange with the surrounding medium. An interconnected network is formed by actin filaments and proteinoid microspheres with tight junctional connections, allowing rapid transmission of thermal-induced conformational changes throughout the structure. This could enable rapid propagation of localized heat stimuli in the composite compared with non-cross-linked individual parts. Additionally, the composite material may allow for new chemical changes and interactions between the natural and artificial components, which can affect the ionization levels of amino acid or peptide side chains engaged in conducting processes. Interfacial chemical effects may alter the opening rates or conductance levels of thermally–activated conduction pathways concentrated at actin–proteinoid binding sites. The larger interface area of the composite increases the potential for adjustable conductive complexes facilitated by temporary molecular orbital overlaps between models. The composite interior likely contains a variety of microenergetic wells due to the diverse surface chemistry and asymmetric geometries in close proximity. This matrix could facilitate complex multi–conformational transitions driven by thermal factors. The simultaneous existence of charge migratory channels with varying sizes and shapes may enable sequential conductive sweeps that add up over time to produce faster-amplified voltage deflections.

Further investigation is needed to understand the specific mechanisms that enhance conductance in the interconnected actin and proteinoid structures. There are numerous potential factors that could be influencing this phenomenon. These factors involve temporary ion transition incidents, the unfolding of conductive protein structures, and changes in the structuring of water at its interfaces. As shown in Table 5, these factors, while difficult to understand on their own, may work together to create a complex synergy. This collaboration leads to heightened sensitivity. Moreover, the composite structure of these materials provides versatility for modulation. This is achieved by intentionally incorporating elements that react to different stimuli. These additions could have a substantial effect on the mechanisms mentioned above. For example, incorporating carbon-based transportation systems can trigger a sequence of electron transfers through the activated channels. On the other hand, incorporating chiral chromophores could lead to the transformation of photonic excitation into transitions of conductive states. It is advantageous to methodically change the additional resources used among a range of architectures. This method can uncover the connections between structure and function, as changes in the former could result in a decrease in the latter, helping to pinpoint optimal combinations and geometric arrangements. This would guarantee the maximization of spike activity triggers. Ultimately, employing a variety of materials and leveraging the interaction of minor energy disruptions could offer a strategy to improve bioelectronic structures. These enhancements would enable these structures to handle more intricate signaling operations effortlessly.

**Table 5:** Mechanisms and Materials for Tailored Proteinoid–Actin Spike Composites

mechanism	description	integratable materials
ion migration	thermal stimulation enables migration of charge carriers across transient tunnels formed at proteinoid–actin interfaces	carbon nanotubes, graphene^97−99^
protein folding	local heating induces unfolding of protein secondary structures, exposing new conductive states	cholesterol, lipids^100−102^
water dynamics	heat alters hydrogen bonding networks surrounding proteins, changing solvation shell tunneling barriers	hydrogels, metal–organic–frameworks^103−105^
photonics	light absorption by composites activates conductive transitions in electronic/vibrational states	chromophores, fluorescent labels^106−108^
magnetics	oscillating magnetic fields can flip spin states of paramagnetic centers tuned at interfaces	iron oxide nanoparticles^109−111^

The results demonstrate conductive behaviors in synthetic matrices driven by physical factors without metabolic support, based on Tamagawa’s hypothesis that the generation of the action potential is a fundamental biological activity.^112^ Nevertheless, the current observations of stimulus-responsive spiking in synthetic actin–proteinoid composites indicate that this behavior may result from self-organized ion adsorption–desorption processes, as opposed to being exclusive to biological systems. At the bionano interface, the composite architecture may establish a variety of reversible ion binding sites and migration pathways. By modulating transient openings and closings of these conductive channels via fluctuations in binding energy, thermal driving forces could enable tunable spike generation as an inherent physicochemical phenomenon. Assuming the combined mixture facilitates more intricate multi-conformational transitions and cooperative conductive paths among charge carrier transient tunneling routes of varying size and shape, this ion adsorption–desorption model would suggest enhanced sensitivity in the composite. The findings, which exhibit action potential-like signals in peptide matrices that are not living, force a reevaluation of long-held assumptions in membrane theory that bioelectrical excitability is solely dependent on active ion transport linked to cellular respiration.

**Figure 14:**
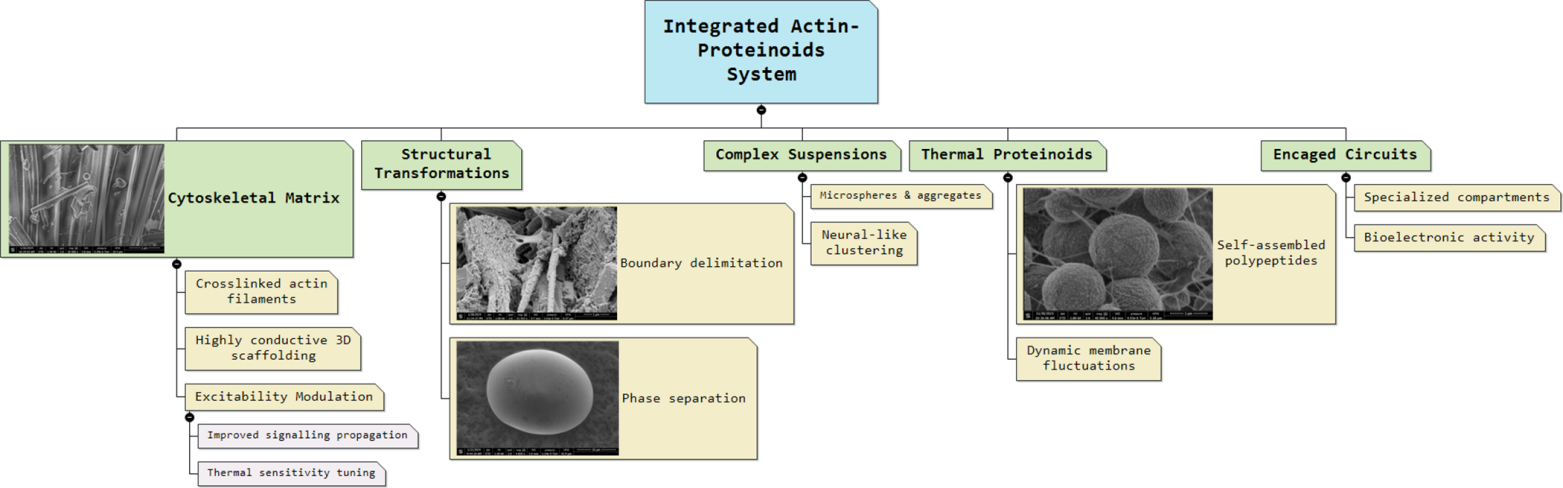
Concept map depicting multiscale mechanism of emergent bioelectronic phenomena caused by interfaced actin–proteinoids systems. Branching hierarchies represent transitory steps between molecular progenitors and sophisticated functional assemblies. The left domain discusses the directed formation of synthetic proteinoids from polymerized amino acids into specialized microspheres with enhanced conductivity. Meanwhile, pure cytoskeletal actin filaments organized into 3D matrices serve as complementing scaffolding elements with inherent signal propagation properties. Integration of these parts at phase borders alters suspension behaviors via delimited topological remodeling and dynamic fluctuations, which regulate collective interactions. Connective overlays highlight composite matrices that extend conduction capabilities beyond individual components. Detailed linkages connect basic structural self-organization to the subsequent tuning of conductive sensitivity at meso/macroscales. The layered landscape picture tries to visualize cross-scale translations, allowing inquiry into guiding naturally derived bioelectronic features for unconventional computing applications by understanding the roots of emergent activity complexities.

By integrating insights obtained from multidimensional structure-function analyses, it is possible to decipher the mechanisms underlying emergent bioelectronic activity in matrices composed of integrated actin filaments and proteinoids. Theoretically depicted in Figure 14 is how molecular-to-mesoscale dynamic coupling regulates the interplay between synthetic and biological systems. Native cytoskeletal filaments build three-dimensional scaffolding structures that are exceptionally conductive and capable of transmitting electronic signals.^113,114^ The implementation of synthetic proteinoids enables the formation of membrane-bound microspheres that spontaneously organize into clustered circuits resembling neural networks.^54,115−117^ Structural transformations arise at phase boundaries, which separate scaffolded networks and self-assembled compartments, due to variations in dynamic bindings. Specified domains serve as a means of restricting and guiding conductivity into specialized circuits for transducing external energy.^118−121^ Localization models the physical connectivity between inputs and outputs in the same way that neural signaling follows protected wire transmission pathways. In the absence of external architectural engineering, this emergent compartmentalized control of information flow increases computational capacities. By comparing pure components and composites using multiparameter analysis, it is possible to control assembly pathways, connectivity patterns, and sensitivity in the direction of bio-inspired adaptive electronics.

Exploring the evolving properties of materials containing proteinoids offers opportunities for bioelectronic devices. Instead of limiting the capabilities of devices with rigid structures, embracing emerging phenomena opens doors to adaptable and life-like platforms. The use of actin filaments as biocompatible frameworks to guide proteinoid aggregation shows potential for integrated biosensing. The ability to capture fluctuating baseline shifts and stimulation sequences enables the conversion of stimulus patterns into an “electronic tissue” without the requirement of specifically engineered transduction elements. Embracing features like the ability to modify thermal sensitivity also provide tools for smart material reshaping in response to external factors. Introducing electronically active nanoparticles or molecular switches inside proteinoid microspheres allows for electro–optical control. The altered protocells may demonstrate regulated signal transmission or responsiveness, offering the fundamental components for adaptive chips. Composite approaches allow for the exploration of novel device physics beyond the constraints of inorganic materials. Exploring the boundary between living organisms and artificial technology presents a novel engineering domain that has not been fully investigated.

The precise manipulation of complex Boolean logic using controlled energy in reactive systems represents a remarkable achievement in collective computation, mimicking aspects of brain networks. The study establishes the ability of interconnected biomolecular networks to represent and change many parameters without requiring dedicated gating systems by systematically describing the combinational logic that generates transient signals.

The findings suggest that complex functions can spontaneously organize in groups of dynamic material that are not in equilibrium, showing life-like flexibility. This suggests unconventional computing opportunities for adaptable morphological modification. Neural circuits provide robust computational ability through interconnected components at many levels. Embracing this distributed intelligence found in these systems presents opportunities for unexplored applications.

## Methods

**Figure 15:**
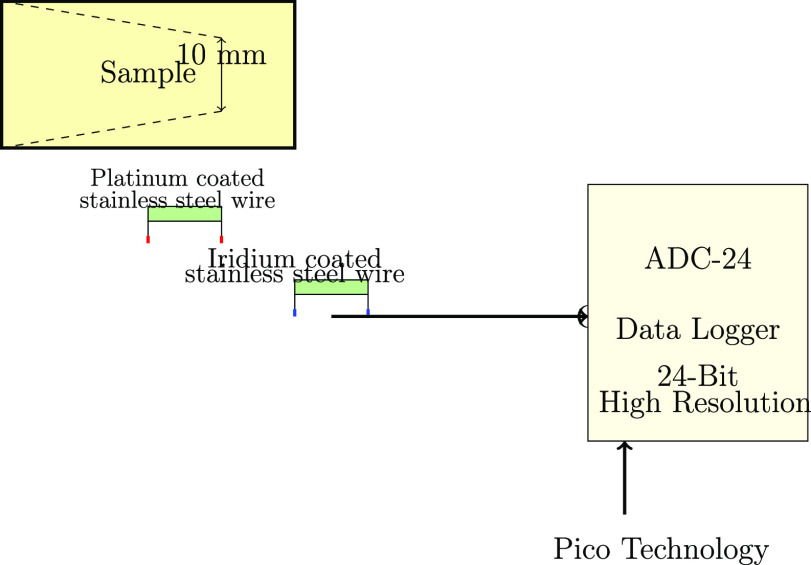
Diagram illustrating the setup for electrochemical measurements. Needle electrodes made from platinum-iridium-coated stainless steel wires were placed 10 mm apart in actin–proteinoid composite samples to map spatiotemporal voltage responses. Signals were obtained using a high-precision 24-bit ADC data logger that was synchronized with a heating block for monitoring thermal and electrical parameters simultaneously. The system has high sensitivity to detect small voltage fluctuations in the μV range.

Purified rabbit skeletal muscle actin was acquired commercially from Cytoskeleton, Inc. The amino acids, such as l-aspartic acid, l-phenylalanine, and l-glutamic acid, were purchased from Sigma-Aldrich without further modifications. Proteinoids were synthesized following previously reported thermal polycondensation techniques,^117^ which involve heating equimolar amino acid mixtures to 180 °C for 30 min under nitrogen. In parallel, a 1% ratio of actin was introduced during this thermal polymerization process to cross-link the formed proteinoids. The resultant composites were isolated via lyophilization to remove unreacted components and stored for characterization. Scanning electron microscopy imaging using a Quanta 650 microscope enabled visualizing the morphological aspects of obtained proteinoid–actin materials following gold coating to increase conductivity for optimal imaging quality. Needle electrodes made from platinum-iridium-coated stainless steel wires were constructed in-house by Spes Medica Srl. and used to record electrical activity from proteinoid–actin mixtures. The electrodes were embedded approximately 10 mm apart within the samples to map propagating responses across this linear distance through the interconnected matrix. Signals were acquired using a high-resolution 24-bit Pico Technology ADC-24 data logger to precisely trace voltage fluctuations with low noise. The electrochemical cell used for measurements is depicted in Figure 15. For specific experiments, samples were also interfaced using an Ossila Instruments T2006A manual potentiostat for open potentiometry recordings following standardized best practice measurement procedures.

Experimental voltage trace measurements were collected from pure proteinoid, actin fiber, and composite samples over 90,012 time steps. The spiking electrical potential profiles were imported into MATLAB 2023b (Mathworks), transformed to fit a 100 × 100 node grid to depict networks of 100,000 stochastic neurons, and color coded to represent quiescent-active-refractory (green–red–black) states similar to the probabilistic forest fire model. The transformation essentially integrates the real-time electrical signal data obtained from 3D electrodes into a 2D spatial framework for visualizing network behavior. Color maps were used to visualize the activity and connectivity patterns of proteinoid, actin, and composite networks at a specific moment during stimulation to highlight unique characteristics. While alternative quantifiers such as wavelet transforms and statistical state sequence analyses provide more robust dynamical profiling, the modified visualizations allow for quick insights into system-level activity changes resulting from additive versus synergistic effects when compared side by side.

The interactions between the proteinoid microspheres and the actin filaments formed a stable network, not fixing the proteinoid–actin composite to the substrate. The actin filaments provided a structural scaffold that held the proteinoid microspheres in place, allowing for the formation of a cohesive network. Repeated measurements over time confirmed the network’s stability, revealing consistent electrical signals without significant fluctuations or degradation. To estimate the number of proteinoid microspheres present in the 10 mm distance between the electrodes, we made the following assumptions: the microspheres are arranged in a single layer, they are tightly packed without overlapping, and the electrodes are parallel with a length much greater than the gap between them. First, we calculated the average diameter of the microspheres (*d*_avg_) based on the given size range of 1–3 μm:
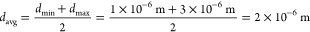
12Next, we approximated the area occupied by a single microsphere (*A*_sphere_) using the average diameter:
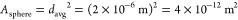
13To determine the total area between the electrodes (*A*_total_), we assumed a rectangular shape with a length equal to the gap between the electrodes (*l*_gap_ = 10 mm) and a width equal to the length of the electrodes (*l*_electrode_), which was assumed to be 5 mm:
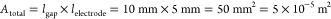
14Finally, we estimated the number of microspheres (*N*_spheres_) by dividing the total area by the area occupied by a single microsphere:
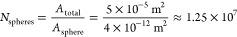
15Based on these assumptions and calculations, we estimate that approximately 1.25 × 10^7^ proteinoid microspheres are present in the 10 mm distance between the electrodes. It is important to note that this is a rough estimate, and the actual number of microspheres may vary depending on factors such as the packing density, size distribution, and the presence of actin filaments in the composite.

**Figure 16:**
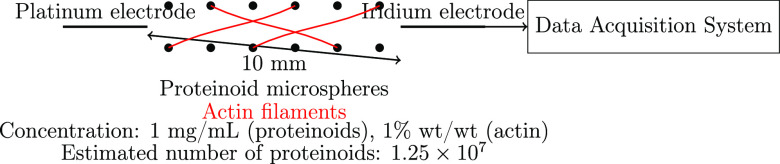
Schematic representation of the experimental setup for measuring the electrical properties of the proteinoid–actin composite. Proteinoid microspheres (1 mg/mL) and actin filaments (1 wt %/wt) are mixed and placed between platinum and iridium electrodes spaced 10 mm apart. The actin filaments form a cross-linked network that interconnects the proteinoid microspheres, creating a stable composite structure. The estimated number of proteinoid microspheres between the electrodes is 1.25 × 10^7^. The electrical signals from the composite are recorded using a data acquisition system.

An enlargement of the experimental setup for measuring the electrical properties of the proteinoid–actin composite is illustrated in Figure 16. Proteinoid microspheres (1 mg/mL) and actin filaments (1 wt %/wt) were mixed and placed between platinum and iridium electrodes spaced 10 mm apart. The actin filaments formed a cross-linked network that interconnected the proteinoid microspheres, creating a stable composite structure. The estimated number of proteinoid microspheres between the electrodes was 1.25 × 10^7^. The electrical signals from the composite were recorded using a PICOLOG data logger with a sampling rate of 1 s and a resolution of 16 bits.

## Conclusions

We have shown the enhancement of thermosensory transient spike activity by a combined biocomposite system of actin filaments cross-linked with proteinoid microspheres. An increase in heating-induced voltage spike sensitivity three times greater than that of individual protein complexes was accomplished. The study uncovers novel sensory responses resulting from interactions between proteins and highlights strategies for improving biosensors by integrating biological elements to enhance detection performance and utilizing cofactors to boost sensitivity to stimuli. Additional research is needed to confirm enhanced sensitivity in cellular thermogenesis pathways and composite-based biosensor technologies using bio-interfaces. Combining proteinoid components may enhance sensory systems crucial for perceiving environmental threats.

## Data Availability

This data is accessible via the online database Zenodo (https://zenodo.org/records/10777713).
